# SPT6 maintains epidermal homeostasis by inhibiting an NF-κB-positive feedback loop to prevent excessive inflammation

**DOI:** 10.1038/s41423-026-01410-1

**Published:** 2026-04-01

**Authors:** Yushuang Sun, Shuqia Xu, Dongyu Wang, Shuiying Gao, Xiaowen Qi, Miao Jiang, Dan Jian, Yaqiong Li, Zhaoyan Jiang, Miao Zhen, Peng Wang, Bin Shu, Julin Xie, Demeng Chen, Qing Tang, Jingting Li

**Affiliations:** 1 Department of Plastic Surgery, Institute of Precision Medicine, The First Affiliated Hospital, Sun Yat-sen University, Guangzhou, China; 2 Department of Obstetrics and Gynecology, The First Affiliated Hospital, Sun Yat-sen University, Guangzhou, China; 3 Department of Burns, The First Affiliated Hospital, Sun Yat-Sen University, Guangzhou, China; 4 Center for Translational Medicine, The First Affiliated Hospital, Sun Yat-Sen University, Guangzhou, China

**Keywords:** SPT6, Skin inflammation, Epidermal differentiation, NF-κB, p65, Mechanisms of disease, Psoriasis

## Abstract

Keratinocytes are increasingly recognized as central regulators of cutaneous immune responses and key contributors to maintaining immune homeostasis. However, whether and how epidermal stem and progenitor cells (EPSCs) actively suppress proinflammatory signaling pathways to prevent excessive inflammation and maintain epidermal immune quiescence remains unclear. Here, we generated a conditional knockout mouse model (K14-CreERT; *Supt6*^fl/fl^) to investigate the role of SPT6, a transcription elongation factor, in epidermal and immune homeostasis. Loss of SPT6 in basal keratinocytes led to spontaneous, psoriasis-like skin inflammation, characterized by epidermal hyperplasia, immune cell infiltration, parakeratosis, and hyperkeratosis. SPT6-deficient mice also exhibited significantly delayed wound healing accompanied by impaired Wnt signaling. Moreover, single-cell RNA sequencing revealed distinct keratinocyte subpopulations with inflammatory signatures, elevated NF-κB signaling, and suppressed Wnt signaling. Mechanistically, SPT6 suppresses NF-κB signaling by binding to an enhancer of the *RELA* gene and preventing its positive transcriptional feedback loop. These findings support a new paradigm in which the default state of the skin may be primed for inflammation, and active suppression by factors such as SPT6 is required to maintain epidermal homeostasis. Taken together, the results of our study reveal a previously unrecognized role for SPT6 as a key regulator of epidermal immune quiescence and tissue integrity.

## Introduction

The skin serves as the body’s first line of defense against environmental insults, and its homeostasis is critical for immune protection. This balance is maintained primarily through the regulation of keratinocyte self-renewal and differentiation, which are key processes for preserving the epidermal barrier. In addition to forming a physical barrier, the epidermis actively participates in immune surveillance. Keratinocytes detect environmental changes and initiate inflammatory responses through the secretion of cytokines, chemokines, and growth factors, positioning them as critical sentinel cells [1]. Disruption of this balance leads to impaired skin barrier function and the development of inflammatory skin diseases, including psoriasis and atopic dermatitis [2–4]. These conditions are characterized by abnormal epidermal differentiation and highlight the importance of epithelial–immune interactions in disease pathogenesis. Therefore, understanding the mechanisms that regulate skin inflammation is crucial for the development of novel therapeutic approaches.

Previous studies from our laboratory and others have demonstrated that transcription factors (TFs) and epigenetic regulators are essential for epidermal homeostasis, with increasing recognition of their roles in maintaining epidermal self-renewal and differentiation [5–9]. Master regulators, such as p63, play pivotal roles across the basal and suprabasal layers of epidermal cells. However, the specific contributions of undifferentiated and differentiated keratinocytes to inflammatory skin diseases remain poorly understood. Our recent work suggests that undifferentiated basal keratinocytes, which express high levels of pattern recognition receptors (PRRs), may drive stronger inflammatory responses than differentiated keratinocytes do. The differentiation-associated TF ZNF750 dampens these responses in differentiated keratinocytes by recruiting LSD1/KDM1A to silence PRR genes [10]. Despite these advances, the full role of basal keratinocytes in immune surveillance and inflammation during epidermal homeostasis is still unclear. It is also uncertain whether the skin is naturally predisposed to inflammation and which regulatory factors are required to maintain this state and epidermal homeostasis.

Here, we demonstrate that epidermal loss of SPT6 (the protein encoded by *SUPT6H* in humans and *Supt6* in mice) disrupts immune homeostasis and induces spontaneous psoriasis-like inflammation. Epidermal conditional *Supt6* knockout (KO) mice exhibit hyperplasia, parakeratosis, hyperkeratosis, immune cell infiltration, and elevated expression of proinflammatory genes, including *Il1b*, *Ccl3*, *S100a8*, and *S100a9*. Transcriptomic analysis revealed enrichment of TNF and IL-17 signaling pathways, which closely resemble the signatures observed in both a mouse psoriasis model (treated with imiquimod) and a human psoriasis model. Interestingly, these phenotypes are microbiota independent. Additionally, *Supt6*-KO mice also display delayed wound healing and reduced Wnt signaling. Single-cell RNA-Seq (scRNA-Seq) analysis revealed the expansion of inflammatory basal and intermediate keratinocyte subpopulations, with suppressed Wnt signaling and heightened NF-κB activity. We demonstrate that SPT6 suppresses NF-κB signaling by directly binding to an enhancer of the *RELA* gene (encoding p65) and blocking its positive transcription feedback loop. These results suggest that SPT6 actively inhibits the NF-κB pathway to prevent autoinflammation of the skin. Together, our results support a new paradigm in which the default state of the skin may be primed for inflammation and highlight SPT6 as a critical suppressor of epidermal immune homeostasis, as it represses NF-κB-driven inflammation to safeguard skin immunity.

## Results

### Construction of *Supt6* conditional knockout mice

**Fig. 1:**
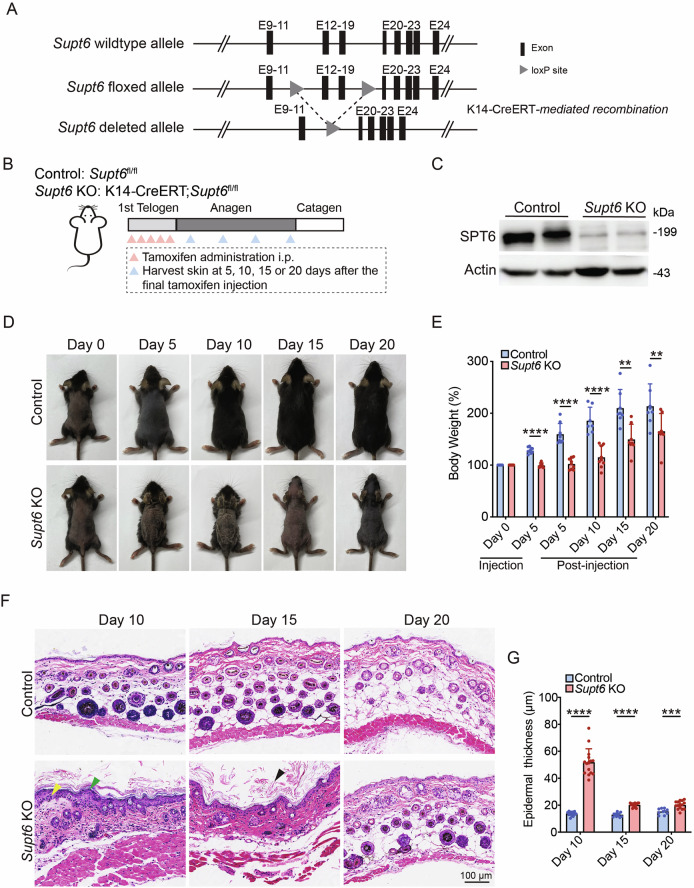
Epidermal deletion of SPT6 delays the hair cycle and induces hyperplasia and hyperkeratosis in the epidermis. **A** Schematic representation of the strategy used to generate conditional *Supt6* knockout (KO) mice using CRISPR-based genetic ablation and the Cre-loxP system. The gray triangles indicate the loxP site flanking the excised exons (black rectangles) of the *Supt6* gene, enabling tissue-specific deletion upon K14-Cre recombinase expression. **B** Diagram illustrating the experimental timeline of tamoxifen-induced SPT6 deletion *via* intraperitoneal injection and the corresponding timepoints for harvesting skin samples. **C** Western blot analysis of SPT6 protein expression in control and *Supt6*-KO dorsal skin harvested on day 10 after the final tamoxifen injection. **D** Representative images showing the gross appearance of control and *Supt6*-KO mice at 0, 5, 10, 15, and 20 days after the final tamoxifen injection. **E** Relative body weight change (%) of control and *Supt6*-KO mice at 0 and 5 days after the first tamoxifen injection and at 5, 10, 15, and 20 days after the final tamoxifen injection. Body weight changes are calculated as percentages relative to the initial weight at injection day 0. **F** H&E staining of dorsal skin from control and *Supt6*-KO mice was performed at 10, 15, and 20 days after the final tamoxifen injection. The yellow arrowhead indicates epidermal hyperplasia. The green arrowheads indicate parakeratosis. The black arrowhead indicates hyperkeratosis. The scale bar represents 100 μm for H&E staining. **G** Quantification of the epidermal thickness of control and *Supt6*-KO mice was performed on days 10, 15, and 20 after the final tamoxifen injection. The mean values are shown with error bars representing the standard deviation (SD). Statistical significance is indicated as follows: **p < 0.01, ***p < 0.001, and ****p < 0.0001. *n* ≥ 6 per group (t-test)

To generate a *Supt6* conditional knockout (KO) mouse model, we engineered mice with floxed *Supt6* alleles in which loxP sites flanked exons 12-19. These mice (*Supt6*^fl/fl^) were then crossed with Keratin 14 (K14)-CreERT transgenic mice [11] to obtain tamoxifen-inducible, tissue-specific K14-CreERT; *Supt6*^fl/fl^ mice (Fig. 1A). Throughout the manuscript, K14-CreERT; *Supt6*^fl/fl^ will be referred to as *Supt6* KO unless otherwise specified.

To induce functional loss of SPT6, 50 mg/kg tamoxifen was administered intraperitoneally once daily for five consecutive days during the first telogen phase to induce SPT6 deletion. Control mice (*Supt6*^fl/fl^) received the same dose of corn oil as the vehicle control did. Skin samples were harvested at 5, 10, 15, and 20 days after the final tamoxifen or corn oil injection (Fig. 1B). Efficient tissue-specific deletion of SPT6 was confirmed at both the DNA and protein levels. Genomic PCR detected the expected floxed (~618 bp) and recombined (~457 bp) *Supt6* alleles specifically in K14-expressing epithelial tissues (Supplementary Fig. 1A). Efficient deletion was further validated by reduced SPT6 protein levels in dorsal skin samples at 10 days post-tamoxifen injection (Fig. 1C).

### Loss of SPT6 disrupts epidermal homeostasis in mouse skin

Notably, within 5 days of initiating tamoxifen treatment, the skin lesions of the *Supt6*-KO mice began to develop, with prominent skin thickening, particularly in the ear. By day 10 post-tamoxifen injection, the dorsal skin of the *Supt6*-KO mice exhibited scaling and crusting, which progressed to peeling and alopecia by day 15. These phenotypes were often accompanied by weight loss and signs of emaciation in more severe cases (Fig. 1D, E). To exclude abnormalities in the K14-positive epithelium of the upper digestive tract, we performed hematoxylin and eosin (H&E) staining of the tongue and esophagus, which revealed intact epithelial architecture with no evidence of epithelial erosion, structural abnormalities, or inflammatory infiltration in the *Supt6*-KO mice compared with the control mice (Supplementary Fig. 1B). The severity of skin lesions varied between mice and correlated with overall survival, depending on the extent and progression of skin pathology. Histological analyses of dorsal skin sections from *Supt6*-KO mice using H&E staining revealed significant disruption of epidermal homeostasis. Key pathological features included detachment of the stratum corneum, parakeratosis, and hyperkeratosis (Fig. 1F).

Additional findings included epidermal thickening and delayed hair cycle progression in *Supt6*-KO mice. Quantification revealed a significant 3-fold increase in *Supt6*-KO epidermis thickness compared with that of control mice at 10 days post-tamoxifen injection (Fig. 1G). Hair follicle analysis revealed preserved staining of the hair follicle stem cell marker SOX9 with no cleaved caspase-3 staining at the telogen phase (day 0 after tamoxifen injection), indicating that the follicle structure remained intact without loss of apoptosis. However, compared with control follicles, early anagen (day 4 after tamoxifen injection) *Supt6*-KO follicles exhibited reduced proliferation (few Ki67-positive cells), indicating impaired activation of hair follicle stem cells in the early anagen phase (Supplementary Fig. 1C). Interestingly, Ki67 staining in the epidermis began to increase on day 7 after the final tamoxifen injection in the *Supt6*-KO mice, which coincided with the onset of epidermal hyperplasia (Supplementary Fig. 1D, E). Both the epidermal thickening and the hair cycle delay were reversed by day 20 after the final injection of tamoxifen in the *Supt6*-KO mice (Fig. 1F, G).

**Fig. 2:**
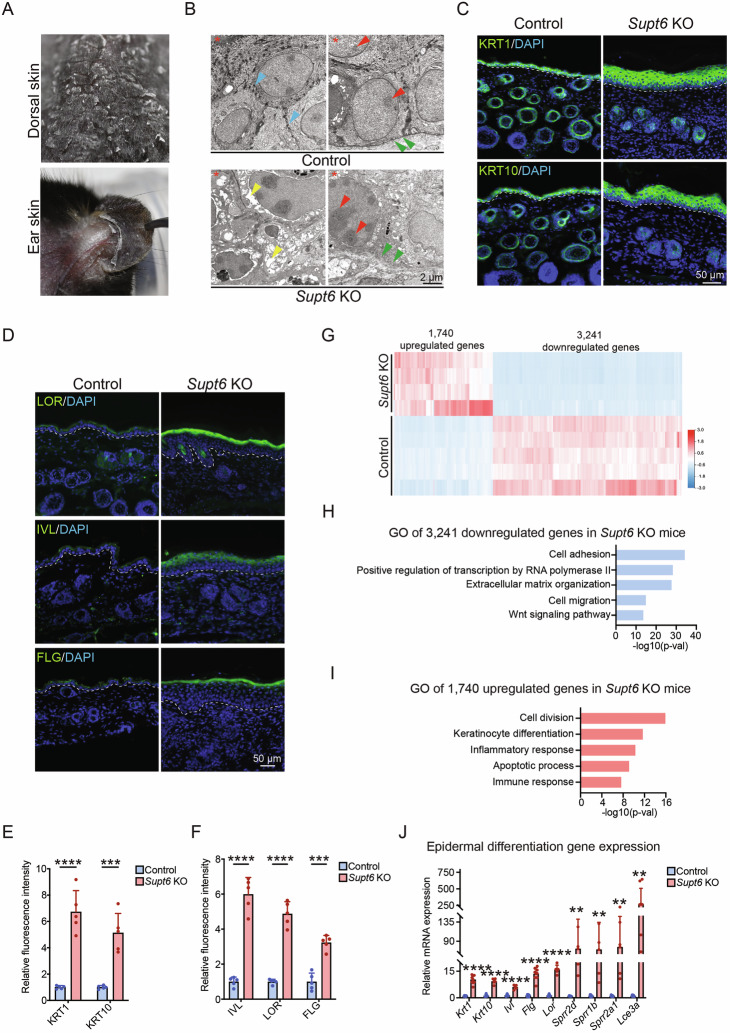
*Supt6* knockout enhances epidermal differentiation. **A** Representative images of dorsal and ear skin from *Supt6*-KO mice at 10 days after the final tamoxifen injection. **B** Transmission electron microscopy (TEM) of dorsal skin harvested from control (top panel) and *Supt6*-KO (bottom panel) mice on day 10 after the final tamoxifen injection. The blue arrowheads indicate desmosomes (top left panel), the yellow arrowheads indicate bubbles (bottom left panel), the green arrowheads indicate basement membrane (top and bottom right panel), the red arrowheads indicate nucleoli (top and bottom right panel), and the red asterisk indicates the epidermal side of the skin. The scale bar represents 2 μm for the TEM images. **C** Immunostaining of Keratin 1 (KRT1) and Keratin 10 (KRT10) in the dorsal skin of control and *Supt6*-KO mice harvested 10 days after the final tamoxifen injection. The white dashed line denotes epidermis/dermis boundaries. The scale bar represents 50 μm for immunostaining. **D** Immunostaining of Involucrin (IVL), Loricrin (LOR), and Filaggrin (FLG) in the dorsal skin of control and *Supt6*-KO mice 10 days after the final tamoxifen injection. The white dashed line denotes epidermis/dermis boundaries. The scale bar represents 50 μm for immunostaining. Quantification of the fluorescence intensity of KRT1/KRT10 (**E**) and IVL/LOR/FLG (**F**). **G** RNA-Seq analysis of control (*n* = 5) and *Supt6*-KO (*n* = 4) skin harvested 10 days after the final tamoxifen injection. Heatmap showing 1740 upregulated (red) and 3241 downregulated (blue) genes on a log2 scale in *Supt6*-KO skin. The color key from blue to red indicates low to high expression levels. **H** Top enriched Gene Ontology (GO) terms for the 3241 downregulated genes according to Enrichr. **I** Top enriched GO terms for the 1740 upregulated genes according to Enrichr. **J** RT‒qPCR analysis of epidermal differentiation genes in control and *Supt6*-KO dorsal skin harvested 10 days after the final tamoxifen injection. The mean values are shown with error bars representing the SDs. Statistical significance is indicated as follows: **p < 0.01, ***p < 0.001, and ****p < 0.0001 (t-test). *n* ≥ 5 per group. All individual dots in the bar graphs represent data from an individual mouse

Phenotypically, the pathological features of the *Supt6*-KO mice were not confined to the dorsal skin but were also observed at other lesion sites, including the ear, palm, and perianal areas (Fig. 2A and Supplementary Fig. 1F). H&E staining of the ear and digital skin confirmed the same histopathological features as those observed in the dorsal skin (Supplementary Fig. 1G).

Transmission electron microscopy (TEM) revealed multiple ultrastructural abnormalities in the *Supt6*-KO epidermis, including increased epidermal thickness, loss of desmosomes and hemidesmosomes, accumulation of keratohyaline granules, basement membrane disruption, cytoplasmic vacuolation, and increased nucleoli (Fig. 2B and Supplementary Fig. 2A). Quantitative analysis confirmed these observations (Supplementary Fig. 2B), suggesting that epidermal hyperplasia, compromised cell adhesion, and altered differentiation occurred in the *Supt6*-KO mice. These findings indicate that the loss of SPT6 compromises epidermal structural integrity and barrier organization, contributing to the observed skin pathology.

Interestingly, when SPT6 deletion was induced before the second telogen in mice, no obvious external skin changes were observed (Supplementary Fig. 2C, D). However, H&E staining of dorsal and ear skin still revealed similar abnormalities, including epidermal thickening and stratum corneum defects, which is consistent with earlier time point findings (Supplementary Fig. 2E). These results suggest that SPT6 is essential for maintaining epidermal homeostasis throughout postnatal skin development.

### *Supt6*-KO mice display enhanced epidermal differentiation

To evaluate how deletion of SPT6 affects epidermal differentiation, we analyzed the expression patterns and levels of several intermediate and terminal epidermal differentiation markers by immunofluorescence microscopy. The expression of Keratin 1 (KRT1) and Keratin 10 (KRT10) increased in the *Supt6*-KO epidermis on day 10 after the final tamoxifen injection (Fig. 2C, E). Although KRT1 expression gradually decreased over time, it remained elevated in the *Supt6*-KO group compared with the control group (Supplementary Fig. 3A, B). Similar increases in KRT1 and KRT10 expression were also observed in the ear and tail skin of *Supt6*-KO mice (Supplementary Fig. 3C–F). Additionally, we detected significant increases in Loricrin (LOR) expression in the dorsal, ear, tail, and tongue skin and increased Involucrin (IVL) and filaggrin (FLG) expression in the dorsal, ear, and tail skin of *Supt6*-KO mice on day 10 (Fig. 2D, F and Supplementary Fig. 3G–N). Consistently, a marked increase in epidermal thickness and elevated expression of KRT1 and LOR were also observed in the dorsal and ear skin of adult *Supt6*-KO mice (Supplementary Fig. 4A–D).

To gain deeper insights into the molecular alterations induced by SPT6 loss, we performed RNA sequencing (RNA-Seq) on lesional dorsal skin from *Supt6*-KO mice and matched dorsal skin from control mice, which were harvested on day 10 after the final tamoxifen injection. Differential gene expression analysis revealed 1740 upregulated genes and 3241 downregulated genes in *Supt6*-KO skin, with a cutoff of ≥ 2-fold change and a *p* value < 0.05 (Fig. 2G and Supplementary Table 1). Gene Ontology (GO) analysis revealed that the 3241 downregulated genes were significantly enriched for pathways related to cell adhesion, ECM organization, and the Wnt signaling pathway. In contrast, the 1740 upregulated genes were enriched in biological processes, including cell division, keratinocyte differentiation, and the inflammatory response, which is consistent with the pathological phenotypes observed in the *Supt6*-KO mice (Fig. 2H, I). We further validated the RNA-Seq results by confirming the significantly increased epidermal differentiation-associated mRNA expression of *Krt1*, *Krt10*, *Ivl*, *Flg*, *Lor*, *Sprr2d*, *Sprr1b*, *Sprr2a1*, and *Lce3a* in *Supt6*-KO skin compared with that in control skin (Fig. 2J).

### Delayed wound healing in *Supt6*-KO mice is associated with suppressed Wnt signaling

**Fig. 3:**
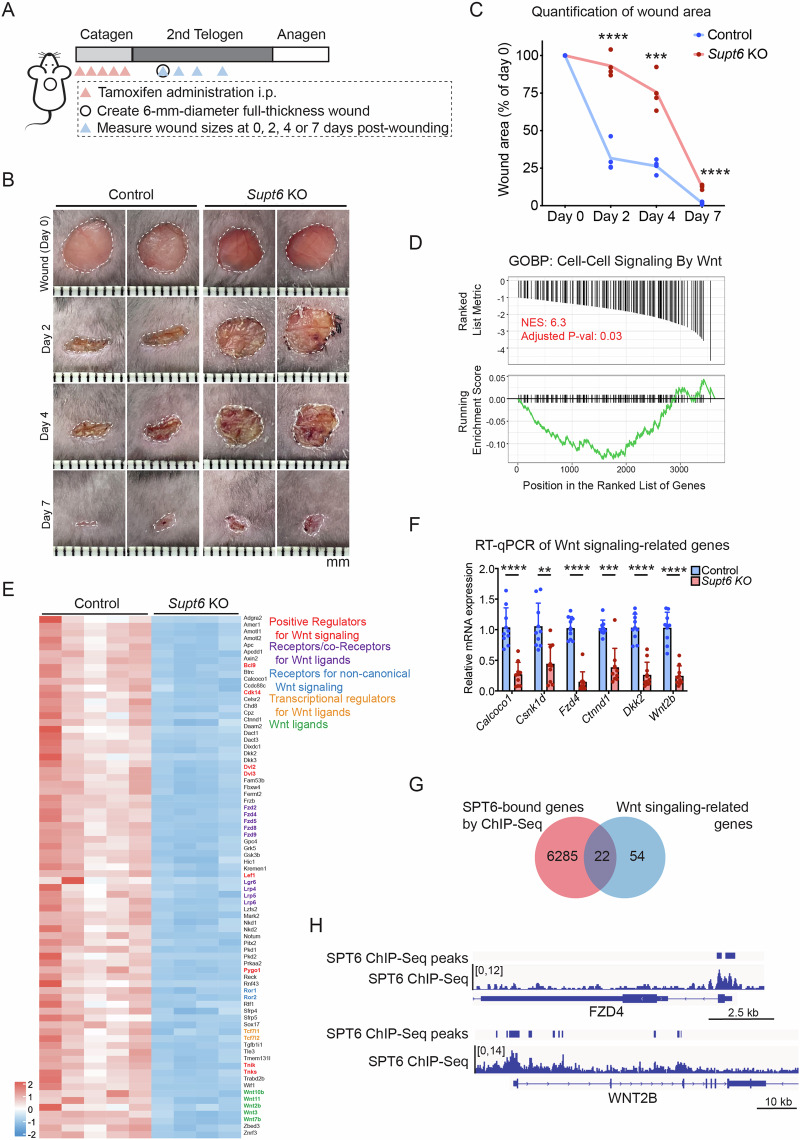
*Supt6* knockout delays wound healing by inhibiting the Wnt signaling pathway. **A** Schematic diagram of tamoxifen-induced SPT6 deletion, wound creation, and wound size measurement time points in control and *Supt6*-KO mice. **B** Representative wound images of control and *Supt6*-KO mice at 0, 2, 4, and 7 days post-wounding. **C** Quantification of the wound area expressed as a percentage of the initial wound size on day 0, as measured by ImageJ (*n* = 4). **D** GSEA indicating downregulation of cell–cell signaling by Wnt in *Supt6*-KO skin. NES = 6.3, adjusted *p* value at 0.03. **E** Heatmap of the expression of 76 genes related to Wnt signaling in control and *Supt6*-KO skin on a log2 scale. **F** RT‒qPCR validation of the reduced expression of Wnt signaling-related genes in the dorsal skin of *Supt6*-KO mice 10 days after the final tamoxifen injection. **G** Venn diagram showing the overlap between SPT6-bound genes according to ChIP-Seq data (red circle) and Wnt signaling-associated genes downregulated upon *Supt6*-KO, as shown in Fig. 3E (blue circle). **H** Gene tracks of SPT6 ChIP-Seq (shown in blue) showing SPT6 binding at the transcription start site of *FZD4* and *WNT2B*. The y-axis shows reads per million, and the blue bars over the gene track represent significant peaks. The mean values are shown with error bars representing the SDs. Each dot represents a single replicate or data from an individual mouse. Statistical significance is indicated as follows: **p < 0.01, ***p < 0.001, and ****p < 0.0001 (t-test). *n* ≥ 6 per group

Given the essential role of SPT6 in maintaining epidermal homeostasis and the close interplay among inflammation, wound healing, and epidermal function, we next investigated whether SPT6 deletion affects skin wound healing. To address this, we generated a full-thickness excisional wound (6 mm in diameter) on the dorsal skin of control and *Supt6*-KO mice on day 5 after the final injection of tamoxifen during the 2nd telogen phase of the hair cycle. Wound sizes were monitored and quantified on days 0, 2, 4, and 7 postwounding (Fig. 3A). As expected, compared with control mice, *Supt6*-KO mice exhibited a significantly delayed wound healing response at all measured time points (Fig. 3B, C). These results indicate that SPT6 is essential for efficient re-epithelialization and proper wound repair in the skin.

Wnt signaling is well known as a critical regulator of the wound healing process [12]. Consistent with these findings, gene set enrichment analysis (GSEA) of the previously mentioned transcriptomic data revealed significant downregulation of Wnt signaling pathways in *Supt6*-KO skin (Fig. 3D). A total of 76 Wnt-related genes, including key positive regulators of Wnt signaling, such as Wnt ligands, receptors, and coreceptors for Wnt ligands, noncanonical Wnt signaling receptors, and transcriptional regulators involved in Wnt signaling, were found to be dysregulated upon SPT6 deletion (Fig. 3E). These findings were further validated by RT‒qPCR, which confirmed the significant downregulation of crucial Wnt signaling regulators, including *Calcoco1*, *Csnk1d*, *Fzd4*, *Ctnnd1*, *Dkk2*, and *Wnt2b* (Fig. 3F). Interestingly, our SPT6 ChIP-Seq data [13] generated from human keratinocytes demonstrated direct binding of SPT6 to 22 out of 76 regulatory genes associated with Wnt signaling, including *FZD4* and *WNT2B* (Fig. 3G, H). These observations suggest that SPT6 may directly regulate the transcription of Wnt pathway genes through promoter occupancy in the epidermis and that loss of SPT6 disrupts Wnt signaling, impairing the wound healing response.

### Transcriptome profiling of *Supt6*-KO skin reveals psoriasis-like inflammatory signatures

**Fig. 4:**
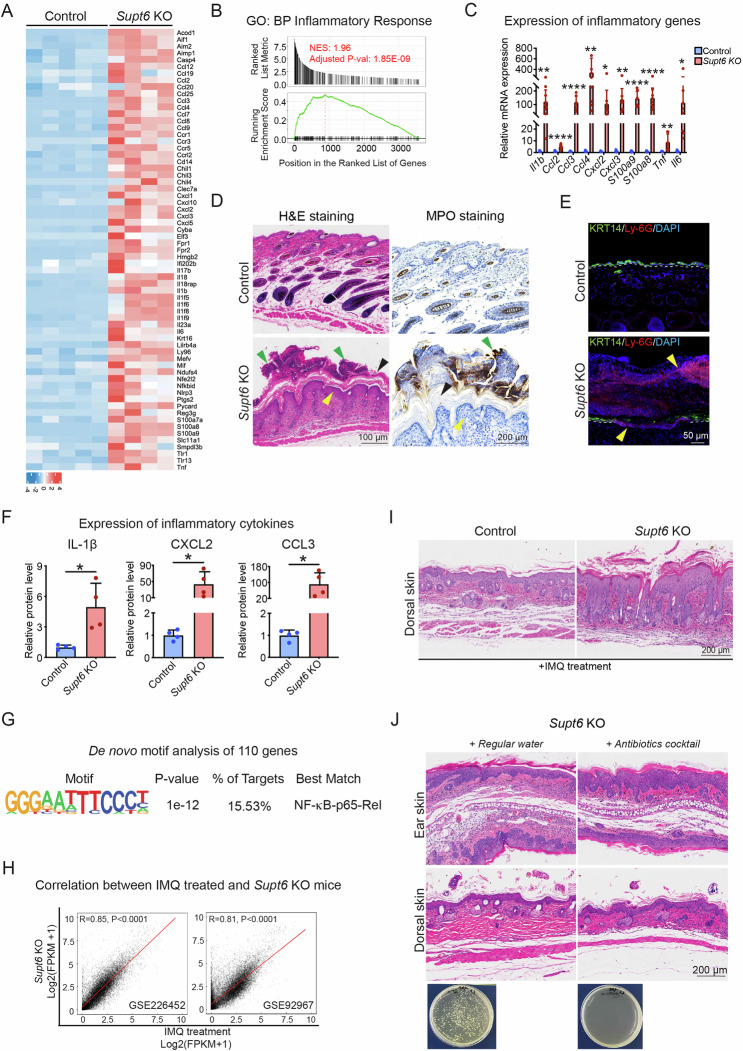
Loss of SPT6 leads to upregulation of inflammatory gene expression. **A** Heatmap displaying the expression of 65 inflammatory genes in control and *Supt6*-KO skin on a log2 scale. The color key from blue to red indicates low to high expression levels. **B** GSEA showing upregulation of the inflammatory response in *Supt6*-KO skin. NES is 1.96, adjusted *p* value is 1.85E-09. **C** RT‒qPCR analysis of inflammatory gene expression in control and *Supt6*-KO skin harvested 10 days after the final tamoxifen injection. **D** H&E and MPO staining of dorsal skin from control and *Supt6*-KO mice harvested 10 days after the final tamoxifen injection. The yellow arrowhead indicates epidermal hyperplasia. The green arrowhead indicates Munro’s microabscesses. The black arrowhead indicates hyperkeratosis. The scale bar represents 100 μm for H&E staining and 200 μm for MPO staining. **E** Immunostaining of Ly-6G and KRT14 in control and *Supt6*-KO dorsal skin harvested on day 5. Yellow arrowheads indicate Ly-6G-positive staining. The scale bar represents 50 μm for immunostaining. **F** Enzyme-linked immunosorbent assay (ELISA) analysis of the inflammatory cytokines IL-1β, CXCL2, and CCL3 in control and *Supt6*-KO skin. Relative protein levels were calculated by dividing the amount of each detected cytokine by the total protein extracted from the tissue and then normalized to that of the control group. **G** De novo motif analysis of 110 upregulated genes associated with the inflammatory response in *Supt6*-KO skin. **H** Pearson correlation analysis between *Supt6*-KO RNA-Seq and two previously published RNA-Seq datasets (GSE226452 and GSE92967) from imiquimod (IMQ)-treated mouse skin. **I** H&E staining of dorsal skin harvested from control and *Supt6*-KO mice 24 h after 3 days of IMQ treatment. **J** H&E staining of ear (top panel) and dorsal skin (middle panel) harvested from control and *Supt6*-KO mice at 10 days after the final tamoxifen injection. Agar plate (bottom panel) shows bacterial colonies cultured from the feces of *Supt6*-KO mice that drank either regular water or antibiotic-containing water, confirming effective microbiota depletion in the antibiotic-treated group. The scale bar represents 200 μm for H&E staining. The mean values are shown with error bars representing the SDs. Each dot in the graph represents a single replicate or data from an individual mouse. Statistical significance is indicated as follows: *p < 0.05, **p < 0.01, and ****p < 0.0001 (t-test). *n* ≥ 4 per group. All individual dots in the bar graphs represent data from an individual mouse

Among the top enriched GO terms, 65 key inflammatory genes, including *Il17b*, *Il1b*, *Il6*, *Il18*, and *Tnf*, which are proinflammatory cytokines known to contribute to psoriasis pathogenesis by promoting keratinocyte activation, neutrophil recruitment, and the Th17-mediated immune response, were identified (Fig. 4A) [14]. To further investigate these transcriptomic changes, we performed GSEA of the differentially expressed genes, which revealed significant enrichment of genes related to the inflammatory response and immune response pathways in the skin of the *Supt6*-KO mice (Fig. 4B and Supplementary Fig. 5A). We further validated the expression of several inflammatory mediators and found significant upregulation of the expression of *Il1b*, *Ccl2*, *Ccl3*, *Ccl4*, *Cxcl2*, *Cxcl3*, *S100a8*, *S100a9, Tnf*, and *Il6* in *Supt6*-KO skin (Fig. 4C). To assess immune cell infiltration, we performed H&E and myeloperoxidase (MPO) staining in the epidermis of control and *Supt6*-KO mice. MPO, a myeloid marker that strongly labels neutrophils, strongly accumulated on the epidermal surface of the dorsal skin of the *Supt6*-KO mice on day 10 after the final injection of tamoxifen, resembling Munro’s microabscesses, a hallmark of psoriatic lesions (Fig. 4D). A similar phenotype was observed in the dorsal and ear skin of adult *Supt6*-KO mice (Supplementary Fig. 5B). We also performed immunostaining for the neutrophil marker Ly-6G in dorsal skin from control and *Supt6*-KO mice harvested on day 5. Ly-6G-positive neutrophils not only accumulated on the surface of the epidermis but also infiltrated the epidermal layer. Intriguingly, this was accompanied by a loss of KRT14 expression in the basal layer of the epidermis in the *Supt6*-KO samples (Fig. 4E). In line with these findings, the protein levels of IL-1β, CXCL2, and CCL3 were also significantly increased in *Supt6*-KO skin (Fig. 4F). Motif enrichment analysis of 110 inflammation-related genes revealed NF-κB-p65-Rel binding motifs (Fig. 4G), suggesting the potential involvement of the NF-κB pathway.

Next, we compared the differential gene expression profiles of the *Supt6*-KO mice with those of two publicly available datasets of IMQ-treated mouse skin [15, 16]. IMQ is a well-established model for psoriasis [17, 18]. We observed a strong positive correlation between *Supt6*-KO lesional skin and IMQ-treated skin (*r* = 0.85 for GSE226452, *p* < 0.0001, and *r* = 0.81 for GSE92967, *p* < 0.0001) (Fig. 4H). Additionally, we detected a moderate positive correlation between the skin of *Supt6*-KO mice and the skin of psoriasis patients (*r* = 0.49 for GSE121212, *p* < 0.0001) (Supplementary Fig. 5C). Among the 1740 upregulated genes in *Supt6*-KO skin, 798 genes (~46%) overlapped with the 2516 upregulated genes on day 6 post-IMQ treatment (GSE92967) (Supplementary Fig. 5D). These overlapping genes were significantly enriched in the IL-17 signaling pathway, NOD-like receptor signaling, cytokine–cytokine receptor, and TNF signaling pathways, as shown by the results of the KEGG pathway analysis (Supplementary Fig. 5E). GO analysis revealed enrichment in biological processes such as keratinocyte differentiation, keratinization, the inflammatory response, and cell cycle regulation (Supplementary Fig. 5F). These findings highlight a partial and conserved inflammatory signature between *Supt6*-KO mice and psoriasis patients, particularly involving genes associated with the inflammatory response, suggesting that this model may be useful for examining inflammation-associated pathways relevant to psoriasis.

We further evaluated the sensitivity of SPT6-deficient skin to inflammatory stimuli by treating both control and *Supt6*-KO mice with IMQ (62.5 mg per day per mouse on a 2 cm × 2 cm dorsal skin) for 3 days. Strikingly, SPT6 deletion significantly exacerbated IMQ-induced psoriasis-like skin alterations, featuring elongated epidermal rete ridges, increased vascularization, abnormal stratum corneum stacking, and scale formation (Supplementary Fig. 5G and Fig. 4I). These findings indicate that the loss of SPT6 sensitizes the skin to IMQ-induced inflammation, further supporting its role in maintaining epidermal immune homeostasis.

To determine whether the skin inflammation observed in *Supt6*-KO mice is caused by secondary bacterial infection due to a compromised epidermal barrier, we administered a broad-spectrum antibiotic cocktail in 1% sucrose drinking water while simultaneously inducing SPT6 deletion for five consecutive days of tamoxifen injection. Dorsal and ear skin samples were collected on days 5 and 10 after the final injection of tamoxifen. Notably, compared with *Supt6*-KO mice given 1% sucrose regular water, *Supt6*-KO mice given antibiotic water developed a skin phenotype, with no apparent differences between the two groups, despite effective bacterial depletion (Fig. 4J). In parallel, periodic acid–Schiff (PAS) staining revealed no evidence of fungal structures in *Supt6*-KO skin (Supplementary Fig. 5H). qPCR analysis of fungal skin scrapings revealed minimal fungal DNA, with no significant differences between control and *Supt6*-KO mice (Supplementary Fig. 5I), and fungal culture on Sabouraud dextrose agar (SDA) and potato dextrose agar (PDA) yielded comparable colony numbers in both groups (Supplementary Fig. 5J). Viral qPCR screening for five common mouse skin viruses, namely, ECTV (ectromelia virus), MPV-1 (mouse parvovirus type 1), mouse papillomavirus (MusPV1/MmuPV1), murine cytomegalovirus (MCMV), and murine gammaherpesvirus 68 (MHV68), revealed only mouse papillomavirus (MusPV1/MmuPV1), with comparable Ct values between groups (Supplementary Fig. 5K). Moreover, to test whether the skin surface microbiota contributes to the phenotype, we performed microbiota transfer experiments by repeatedly swabbing the dorsal skin of control and *Supt6*-KO donors and transferring them to *Supt6*-KO recipients. *Supt6*-KO recipients receiving control or *Supt6*-KO microbiota developed indistinguishable skin phenotypes (Supplementary Fig. 5L). In addition, epidermal RNA-Seq revealed no enrichment of interferon-stimulated gene signatures or pathogen-associated transcriptional responses, suggesting that viral infection was not involved. Collectively, these results indicate that microbial infection is unlikely to be the primary driver of inflammation in *Supt6*-KO mice and support a predominantly cell-intrinsic inflammatory mechanism.

### Single-cell RNA-Seq analysis revealed altered epidermal subpopulations and disrupted differentiation dynamics in *Supt6*-KO mice

**Fig. 5:**
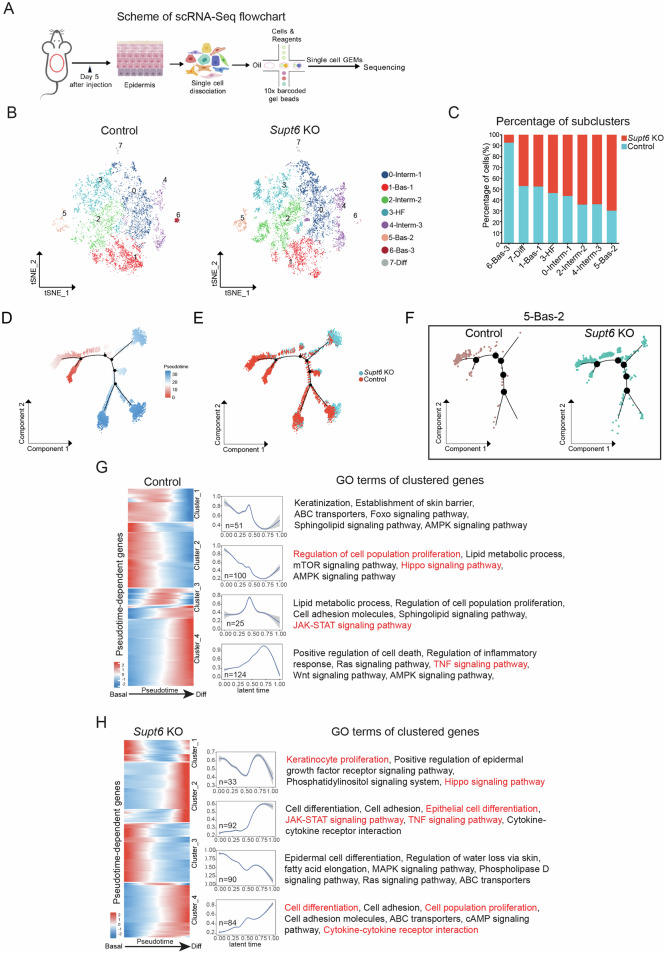
Single-cell RNA-Seq profiling of *Supt6* KO epidermis. **A** Schematic representation of the single-cell RNA sequencing (scRNA-Seq) workflow. A total of 7632 cells from control mice and 10293 cells from *Supt6*-KO mice were retained for downstream analyses. **B** t-Distributed Stochastic Neighbor Embedding (t-SNE) plot of eight keratinocyte subclusters, including three basal (Bas-1, -2, and -3), three intermediate (Interm-1, -2, and -3), one hair follicle subpopulation (HF), and one terminal differentiated (Diff) cell subpopulation. **C** Percentage distribution of keratinocyte subclusters in control (teal) and *Supt6*-KO mice (red). Monocle2 pseudotime trajectory analysis of epidermal keratinocyte subclusters. Cells are ordered along pseudotime from red to blue (**D**) and colored by genotype (**E**). **F** Subset trajectories of subcluster 5 (5-Bas-2) in control and *Supt6*-KO epidermis. **G**&**H** Pseudotemporal gene expression dynamics in control (**G**) and *Supt6*-KO (**H**) epidermis. A heatmap (left) displays the expression of genes along the pseudotime trajectory. Each row represents a gene, normalized to its peak value. The blue to red color gradient indicates low to high gene expression levels. Line plot (middle) shows the smoothed average expression trends of gene clusters over latent time. The number of genes per cluster is indicated in the lower left corner of each plot. The top GO terms (right) enriched in each cluster reflect the dominant biological processes associated with the dynamic expression patterns

To determine how SPT6 depletion in basal keratinocytes alters epidermal subpopulations and promotes inflammatory gene expression, we performed scRNA-Seq on the dorsal epidermis of control and *Supt6*-KO mice on day 5 after the final tamoxifen injection, which corresponds to early phenotype onset (Fig. 5A). After quality filtering, we retained 7632 cells from control mice and 10293 cells from *Supt6*-KO mice for downstream analyses.

Integrated analysis of control and *Supt6*-KO samples using Seurat revealed six major cell types based on canonical marker gene expression: keratinocyte_basal (*Krt14*, *Col17a1*, and *Fcgbp*), keratinocyte_suprabasal (*Krt1*, *Flg*, and *Lor*), dividing cells (*Birc5* and *Pclaf*), hair follicle-associated cells (*Krt17* and *Krt79*), melanocytes (*Pax3* and *Kit*), and immune cells (*Ptprc* and *Cd207*) (Supplementary Fig. 6A, B). As expected, *Supt6* expression was markedly reduced in *Supt6*-KO epidermis, particularly in basal, suprabasal, and dividing keratinocyte populations (Supplementary Fig. 6C), confirming efficient deletion in the major epidermal cell types. Cell type composition analysis revealed increased proportions of dividing cells and keratinocyte_suprabasal cells, with decreased numbers of hair follicle-associated, keratinocyte_basal, and immune cells in the *Supt6*-KO epidermis (Supplementary Fig. 6D). The reduced immune cell proportions on day 5 in the isolated epidermis precluded detailed analysis but indicated that the loss of SPT6 may initiate the keratinocyte-intrinsic inflammation that precedes immune cell recruitment.

Compared with those in the control epidermis, the G2/M score in the *Supt6*-KO epidermis was elevated overall (Supplementary Fig. 6E, F). Specifically, both basal and suprabasal keratinocytes exhibited increased G2/M scores (Supplementary Fig. 6G). Similarly, the proportion of cycling cells (in the G2/M + S phase) increased in both populations, and the expression of the proliferation marker *Cdk4*increased (Supplementary Fig. 6H, I), confirming the enhanced proliferative activity of the *Supt6*-KO keratinocytes.

To minimize the cofounding effects of cell cycle gene expression, the dividing cells were excluded from downstream analyses, as previously recommended [19]. Further subclustering of keratinocytes and hair follicle-associated cells revealed eight distinct populations: three basal (Bas-1, Bas-2, Bas-3), three intermediate (Interm-1, Interm-2, Interm-3), one hair follicle-associated (HF), and one differentiated (Diff) subcluster, each defined by distinct marker gene expression patterns (Fig. 5B and Supplementary Fig. 6J). Quantitative analysis revealed the most prominent increase in the proportions of subclusters 4 (Interm-3) and 5 (Bas-2) and a marked decrease in subcluster 6 (Bas-3) in the *Supt6*-KO epidermis (Fig. 5C). Consistently, the G2/M score was significantly elevated in subclusters 4 and 5 of the *Supt6*-KO epidermis (Supplementary Fig. 6K).

To characterize the dynamic changes in keratinocyte states, we performed pseudotime analysis using Monocle2. Basal keratinocytes expressing canonical progenitor markers (*Itgb1* and *Col17a1*) were designated as the trajectory starting point, whereas terminally differentiated keratinocytes enriched for late differentiation markers (*Lce1b* and *Lce1a1*) were assigned as terminal states. Monocle2 reconstructed a continuous differentiation trajectory from basal progenitors to terminally differentiated keratinocytes, with branching at intermediate stages. Although control and *Supt6*-KO cells exhibited a similar global trajectory topology, the control epidermis showed a gradual and orderly progression transition along pseudotime. In contrast, compared with WT cells, *Supt6*-KO cells displayed a pronounced shift in pseudotime distribution across subclusters, particularly within Bas-2 and Interm-3 (Fig. 5D–F and Supplementary Fig. 7A, B), which is consistent with the enhanced and premature differentiation dynamics observed upon *Supt6* depletion.

To validate the above findings, we performed complementary pseudotime inference approaches. Slingshot analysis independently confirmed a smooth lineage transition from undifferentiated progenitors to terminally differentiated keratinocytes in control cells, whereas the accumulation of Bas-2 and Interm-3 subclusters along the trajectory was similar in *Supt6*-KO cells, despite ultimately reaching terminal differentiated states (Supplementary Fig. 7C). In parallel, CytoTRACE analysis revealed significantly reduced differentiation potential across most *Supt6*-KO subclusters (except Bas-2), indicating decreased transcriptional plasticity and premature commitment toward differentiated fates (Supplementary Fig. 7D, E). Collectively, these complementary analyses demonstrate that SPT6 loss disrupts the coordinated progression of keratinocytes from basal through intermediate differentiation states, resulting in aberrant trajectory dynamics and altered epidermal homeostasis.

To identify key molecular changes during cell state transitions, we performed pseudotemporal dynamics analysis and identified approximately 300 pseudotime-dependent genes in each dataset that clustered into four expression patterns (Fig. 5G, H). Cells were ordered along pseudotime from start to endpoints using the same biological criteria as in the Monocle2 analysis above. Distinct gene expression patterns between the control and *Supt6*-KO epidermis were observed. The expression of cell proliferation-associated pathways, including Hippo signaling, decreased with increasing pseudotime in the control epidermis (Cluster 2), whereas in the *Supt6*-KO epidermis, these pathways exhibited a biphasic pattern, initially decreasing but then increasing (Cluster 1). Conversely, the expression of inflammation-associated pathways, including JAK-STAT and TNF signaling, progressively increased along pseudotime in the *Supt6*-KO epidermis, in contrast to the transient upregulation (increase then decrease) observed in the control epidermis. Similarly, cell differentiation-associated pathways showed sustained upregulation along pseudotime in the *Supt6*-KO epidermis (Cluster 4). These contrasting gene expression patterns along pseudotime suggest that SPT6 loss promotes a transcriptional program characterized by inflammation and aberrant differentiation dynamics.

**Fig. 6:**
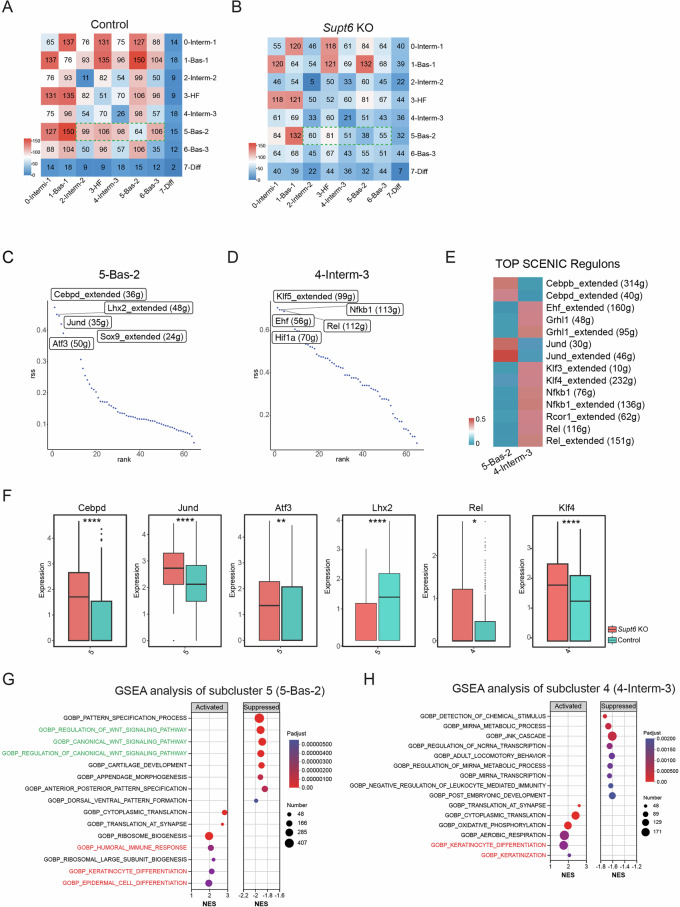
Loss of SPT6 disrupts keratinocyte state transitions and promotes inflammatory transcription factor regulons and pathway signatures in specific subclusters. CellPhoneDB analysis of keratinocyte subcluster interactions in control (**A**) and *Supt6*-KO (**B**) mouse epidermis. The heatmap displays the predicted strength of ligand–receptor–mediated communication between keratinocyte subclusters. The color intensity represents the interaction strength, with red indicating the strongest predicted interactions. Ranked regulon-specific score (RSS) from SCENIC analysis in keratinocyte subclusters 5 (5-Bas-2) (**C**) and 4 (4-Interm-3) (**D**). **E** SCENIC heatmap of transcription factor activity in keratinocyte subclusters 4 and 5. The color key from blue to red indicates low to high expression levels. The number in parentheses represents the number of genes in the regulon. **F** Regulon activity in control (teal) and *Supt6*-KO (red) epidermis across corresponding keratinocyte subclusters. Box plots showing the expression distributions of the *Cebpd*, *Jund*, *Atf3*, *Lhx2*, *Rel*, and *Klf4* regulons. **G** GSEA of genes enriched in keratinocyte subcluster 5 (5-Bas-2). Dot plot summarizing the top activated and suppressed pathways enriched in 5-Bas-2, with the NES on the x-axis. Dot size indicates the number of genes enriched in each pathway, while color reflects statistical significance (adjusted *p* value), with red indicating greater significance. **H** GSEA of genes enriched in subcluster 4 (4-Interm-3). The dot plot displays the top activated and suppressed pathways enriched in 4-Interm-3, with the NES on the x-axis. Dot size indicates the number of genes enriched in each pathway, while color reflects statistical significance (adjusted *p* value), with red indicating greater significance. Statistical significance is indicated as follows: *p < 0.05, **p < 0.01, and ****p < 0.0001 (t-tests were performed for comparisons between two groups)

To characterize the cell‒cell communication landscape between keratinocyte subpopulations, we performed ligand‒receptor interaction analysis using CellPhoneDB 2.0 [20], which predicts enriched interactions from scRNA‒Seq data using a curated repository of ligand‒receptor pairs. This analysis revealed a significant overall reduction in predicted cell‒cell interactions in the *Supt6*-KO epidermis. Notably, subcluster 5 (Bas-2) showed markedly reduced interactions with subclusters 2, 4, and 6 (Fig. 6A, B), which is consistent with the compromised cell adhesion structures observed by TEM analysis and the downregulation of cell adhesion genes revealed by bulk RNA-Seq analysis. Despite global cell‒cell communication reduction, specific pathways showed altered activity in *Supt6*-KO epidermis. Interactions between ephrin ligands (*Efna1*/3) and Eph receptors (*Epha1/2/4*) were upregulated, whereas *App*-*Tnfrsf21* interactions were decreased, particularly in subclusters 4 (Interm-3) and 5 (Bas-2) (Supplementary Fig. 7F, G), suggesting the selective rewiring of intercellular communication networks.

Finally, to identify transcriptional regulators underlying cell fate specification in keratinocyte subclusters 4 and 5, we performed single-cell regulatory network inference and clustering (SCENIC) to infer TF regulon activity on the basis of the coexpression of their downstream target genes [21]. SCENIC analysis revealed enrichment of core regulatory TFs, including *Cebpd*, *Jund*, *Lhx2, Sox9*, *Cebpb*, and *Atf3*, in subcluster 5 (Bas-2) and *Rel*, *Nfkb1*, *Ehf*, *Grhl1*, *Rcor1*, *Klf3*, *Klf4*, and *Hif1a* in subcluster 4 (Interm-3) (Fig. 6C–E). Among these genes, *Lhx2* [22] and *Sox9* [23] are involved in hair follicle and epidermal stem cell regulation. In addition, *Cebpd* [24], *Klf4* [5]*, Klf3* [25]*, Grhl1* [26]*, Ehf* [27], and *Rcor1* [28] promote epidermal differentiation, supporting the phenotypic changes observed in *Supt6*-KO skin. Conversely, *Rel* [29], *Hif1a* [30], *Cebpb* [31], *Jund* [32], and *Nfkb1* [33] are TFs associated with the inflammatory response in the skin, further aligning with the inflammatory gene expression signature detected by bulk RNA-Seq in SPT6-deficient skin.

Notably, the expression of multiple enriched regulons—including *Cebpd*, *Jund*, *Atf3*, *Rel*, *Hif1a*, and *Klf4*—together with their downstream target genes was upregulated in the Interm-3 and Bas-2 subclusters of the *Supt6*-KO epidermis compared with those in the control epidermis, whereas *Lhx2* expression was reduced in Bas-2 in the *Supt6*-KO epidermis (Fig. 6F and Supplementary Fig. 7H). GSEA of the DEGs in these subclusters revealed activation of keratinocyte differentiation, epidermal cell differentiation, and keratinization pathways in the *Supt6*-KO epidermis. Additionally, immune response pathways were significantly activated in subcluster Bas-2 (Fig. 6G, H). These findings corroborated our bulk RNA-Seq results and demonstrated that SPT6 loss drives *the* selective expansion and transcriptional reprogramming of specific basal and spinous keratinocyte subpopulations characterized by enhanced differentiation and inflammatory signatures. Given the prominent upregulation of NF-κB**-**associated regulons and their target genes in these expanded subclusters, we sought to investigate the mechanistic link between SPT6 loss and NF-κB pathway activation.

### SPT6 suppresses inflammatory signaling in keratinocytes *via* the NF-κB pathway

**Fig. 7:**
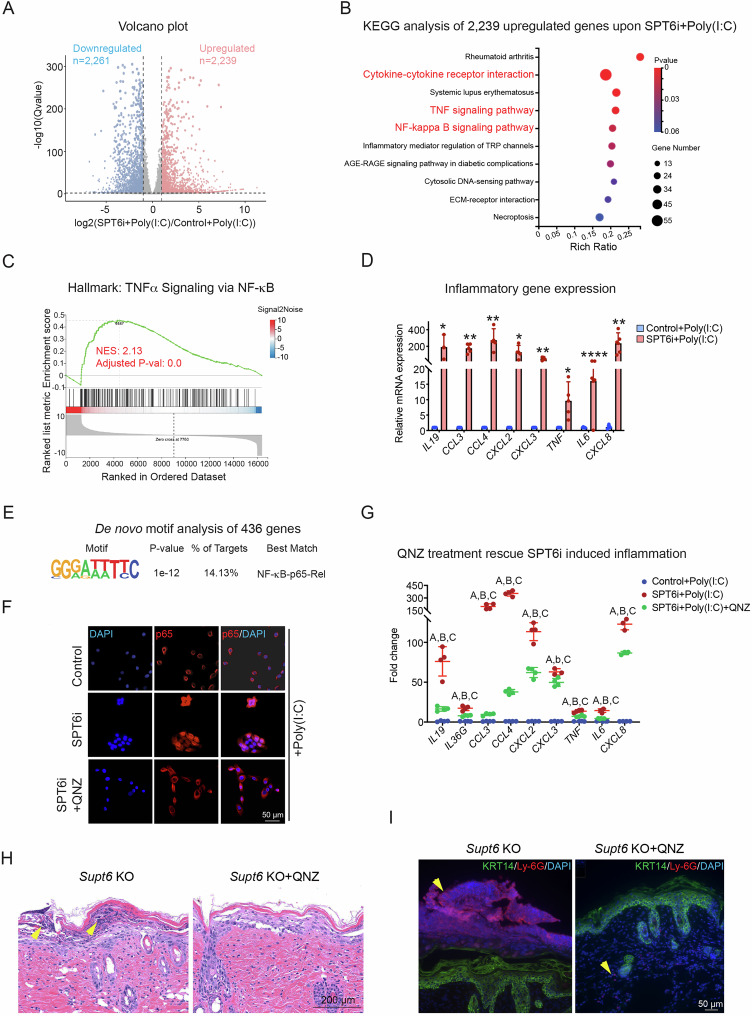
SPT6 suppresses inflammation *via* NF-κB signaling in proliferating human keratinocytes. **A** Proliferating primary human keratinocytes were transfected with scrambled control (Control) or SPT6 (SPT6i) siRNAs (*n* = 2) for 3 days, followed by 4 h of treatment with high-molecular-weight poly(I: C) before being harvested for RNA-Seq. A volcano plot displays the significantly differentially expressed genes when the cutoff was a ≥ 2-fold change and a *p* value ≤ 0.05. **B** KEGG analysis of 2239 upregulated genes in SPT6i- and poly(I: C)-treated keratinocytes. The dot plot displays the top enriched pathways, with the enrichment ratio on the x-axis. Dot size indicates the number of genes enriched in each pathway, while color reflects statistical significance (adjusted *p* value), with red indicating greater significance. **C** GSEA indicating the upregulation of hallmark gene signatures associated with TNF α signaling *via* NF-κB in SPT6i cells. NES at 2.13, adjusted *p* value at 0.0. **D** RT‒qPCR of the inflammatory genes *IL19*, *CCL3*, *CCL4*, *CXCL2*, *CXCL3*, *TNF*, *IL6*, and *CXCL8* in control and SPT6i cells after poly(I: C) treatment. **E** De novo motif analysis of 436 upregulated genes associated with the inflammatory response upon SPT6i + poly(I: C) treatment. **F** Immunostaining of p65 in control cells treated with poly(I: C) or SPT6i cells treated with poly(I: C) alone or in combination with QNZ. The scale bar represents 50 μm for immunostaining. **G** RT‒qPCR analysis of inflammatory gene expression in control cells treated with poly(I: C) and in SPT6i cells treated with poly(I: C) alone or in combination with QNZ. “A” comparison between Control+Poly(I: C) and SPT6i+Poly(I: C). “B” and “b” comparison between SPT6i+Poly(I: C) and SPT6i+Poly(I: C) + QNZ. “C” comparison between Control+Poly(I: C) and SPT6i+Poly(I: C) + QNZ. Capitalized “ABC” indicates p < 0.01, and lowercase “b” indicates *p* < 0.05. **H** H&E staining of dorsal skin harvested from *Supt6*-KO mice 7 days after tamoxifen induction, with or without QNZ treatment. The scale bar represents 200 μm for H&E staining. **I** Immunostaining of Ly-6G and KRT14 in dorsal skin harvested from *Supt6*-KO mice 7 days after the final tamoxifen injection, with or without QNZ treatment. The scale bar represents 50 μm for immunostaining. The mean values are shown with error bars representing the SDs. Each dot in the graph represents a single replicate or data from an individual sample. Statistical significance is indicated as follows: *p < 0.05, **p < 0.01, ***p < 0.001, ****p < 0.0001 (one-way ANOVA followed by Tukey’s multiple comparison for 3 groups, and a t-test was performed for comparisons between two groups). *n* ≥ 4 per group

To elucidate the molecular mechanism by which SPT6 suppresses inflammation in keratinocytes, we performed siRNA-mediated knockdown of SPT6 (SPT6i) in proliferating keratinocytes, followed by polyinosinic-polycytidylic acid (poly(I: C)) stimulation and transcriptomic profiling *via* RNA-Seq. Poly(I: C) is a synthetic analog of double-stranded RNA (dsRNA) and has been widely used to model inflammation caused by tissue damage and viral infection [10, 34]. Compared with control siRNA-treated cells, SPT6i cells treated with poly(I: C) presented 2239 upregulated genes and 2261 downregulated genes (Fig. 7A and Supplementary Table 2). KEGG pathway analysis of the upregulated genes revealed significant enrichment of inflammation-related signaling pathways, including cytokine‒cytokine receptor interactions, TNFα signaling, and the NF-κB signaling pathway (Fig. 7B). GSEA further confirmed the activation of TNFα signaling *via* NF-κB and the inflammatory response in SPT6-depleted cells upon poly(I: C) stimulation (Fig. 7C and Supplementary Fig. 8A). We validated these findings by RT‒qPCR and confirmed robust increases in the expression of key inflammatory genes, such as *IL19*, *CCL3*, *CCL4*, *CXCL2*, *CXCL3*, *TNF*, *IL6*, and *CXCL8*, in SPT6i cells treated with poly(I: C) (Fig. 7D). These results suggest that SPT6 plays a critical role in repressing inflammatory gene expression and maintaining immune homeostasis in human keratinocytes under stress conditions.

To identify transcriptional regulators influenced by SPT6 in the context of inflammatory activation, we first performed de novo motif enrichment analysis on the promoters of 436 inflammation-associated upregulated genes from the SPT6i+Poly(I: C) RNA-Seq dataset. This analysis revealed a significant overrepresentation of NF-κB-p65-Rel binding motifs (Fig. 7E).

To determine whether SPT6 suppresses inflammation through inhibition of the NF-κB signaling pathway, we treated SPT6i cells with 5 μM QNZ, a potent and selective NF-κB inhibitor [35, 36]. Compared with control+poly(I: C) cells, SPT6i+poly(I: C) cells displayed increased nuclear localization of p65, which was markedly reduced upon QNZ treatment (Fig. 7F and Supplementary Fig. 8B). The expression of the inflammatory genes shown in Fig. 7D was also partially rescued by QNZ treatment in SPT6i+Poly(I: C) cells (Fig. 7G). To further confirm that NF-κB signaling was activated in vivo, we administered QNZ to *Supt6*-KO mice for 3 days prior to Cre induction. QNZ treatment alleviated skin inflammation, as evidenced by decreased formation of Munro’s microabscesses and reduced Ly-6G immunostaining in *Supt6*-KO mice on day 7 after the last tamoxifen injection (Fig. 7H, I).

### SPT6 binds to *RELA* at its enhancer region and inhibits its positive feedback loop at the transcriptional level

Intersection analysis between genes upregulated upon SPT6i and poly(I: C) stimulation and those directly bound by SPT6 revealed 721 overlapping genes (Supplementary Fig. 8C). These genes were significantly enriched for GO terms related to the establishment of the skin barrier, skin epidermis development, and positive regulation of the inflammatory response (Supplementary Fig. 8D). KEGG pathway analysis further revealed enrichment of TNFα signaling *via* NF-κB, the UV response, and IL-2/STAT5 signaling (Supplementary Fig. 8E).

**Fig. 8:**
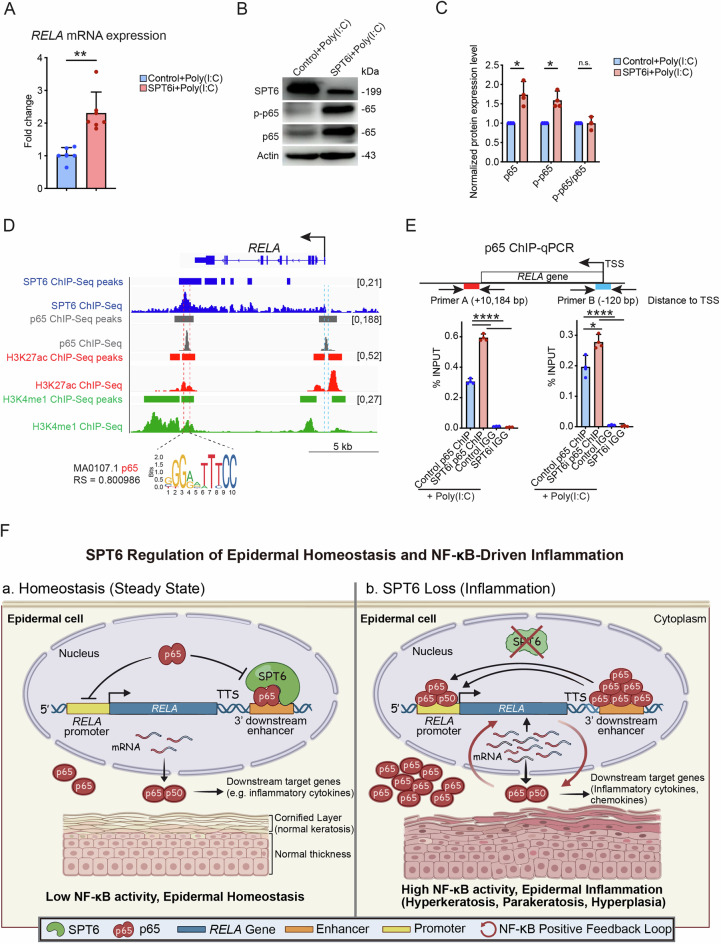
SPT6 inhibits the positive feedback transcriptional activation of *RELA* through binding to its enhancer region. **A** *RELA* mRNA expression in control and SPT6i cells treated with poly(I: C) was measured by RT‒qPCR. **B**, **C** Western blot analysis and quantification of SPT6, p-p65, and p65 expression in control and SPT6i cells treated with poly(I: C). **D** Gene track of the *RELA* gene. The SPT6 ChIP-Seq signal and peaks are shown in dark blue. H3K27ac ChIP-Seq signals and peaks are shown in red. H3K4me1 ChIP-Seq signals and peaks are shown in green. The p65 ChIP-Seq signal and peaks are shown in gray. The y-axis shows the number of reads per million, and the colored bars above the signal tracks denote peak calls. The p65 binding motif predicted by JASPAR (motif ID: MA0107.1; relative score: 0.800986) is displayed at the bottom of the track. **E** ChIP‒qPCR of p65 binding at the *RELA* enhancer and promoter region in control and SPT6i cells treated with poly(I: C). **F** Schematic diagram of the proposed mechanism. The mean values are shown with error bars representing the SDs. Each dot in the graph represents a single replicate or data from an individual sample. Statistical significance is indicated as follows: *p < 0.05, **p < 0.01, ***p < 0.001, and ****p < 0.0001 (one-way ANOVA followed by Tukey’s multiple comparison for 3 groups, and a t-test was performed for comparisons between two groups). *n* ≥ 3 per group

To determine whether p65 expression is elevated in SPT6i cells, we measured changes in overall p65 levels following SPT6 knockdown. This upregulation was validated using RT‒qPCR and western blot analysis (Fig. 8A–C). Next, we investigated whether SPT6 regulates *RELA* (which encodes the p65 protein) transcriptionally by comparing our previously generated SPT6 ChIP-Seq data with publicly available p65 ChIP-Seq data from KB cells, an epithelial cell line [13, 37]. Notably, we detected significant overlap between the binding peaks of SPT6 and p65 at an enhancer region downstream of the *RELA* transcription termination site. This region is marked by H3K27ac and H3K4me1 histone modifications, which are characteristic of active enhancers, as identified in proliferative keratinocytes (ENCODE database). Additionally, motif analysis using JASPAR revealed strong enrichment of the p65 motif within this enhancer region, with a relative score of 0.800986 (Fig. 8D).

On the basis of these findings, we hypothesized that SPT6 regulates p65 binding at this enhancer region to prevent the formation of a positive transcriptional feedback loop. To test this hypothesis, we performed ChIP‒qPCR using a p65 antibody in both control and SPT6i cells treated with poly(I: C). Our results revealed a significant increase in p65 binding at both the promoter and enhancer regions in SPT6-depleted cells (Fig. 8E). Together, these findings support a model in which SPT6 constrains NF-κB-driven transcriptional programs, thereby limiting the magnitude of the inflammatory response in basal keratinocytes (Fig. 8F).

## Discussion

The mechanisms by which epidermal stem and progenitor cells contribute to skin inflammation while preserving epidermal homeostasis remain incompletely understood. In this study, we demonstrate that the loss of SPT6 in the epidermis triggers spontaneous, psoriasis-like inflammation that occurs independently of the microbiota, underscoring a cell-intrinsic mechanism. During tissue repair, SPT6 is essential for maintaining epidermal homeostasis through the maintenance of Wnt signaling, a pathway critical for effective wound healing. scRNA sequencing revealed previously unrecognized heterogeneity within the epidermis, including the emergence of distinct inflammatory subpopulations following SPT6 deletion. Mechanistically, SPT6 functions as a transcriptional gatekeeper, binding to the *RELA* enhancer to prevent NF-κB-driven positive feedback activation, thereby restricting excessive proinflammatory gene expression. These findings reveal a previously unappreciated role for SPT6 as a transcriptional suppressor, in addition to its function as a transcription elongation factor, in safeguarding basal keratinocytes from inflammatory reprogramming.

Recent single-cell analysis studies of inflammatory diseases have largely focused on nonkeratinocyte inflammatory and immune populations, often overlooking the complexity and heterogeneity within the epidermis itself and their potential roles as both sensors and effectors in epithelial immunity [38, 39]. Notably, keratinocytes are the major cell types in the epidermis, and they are not a uniform population. A recent study revealed that ~60% of basal cells respond to *Staphylococcus aureus* (SA) challenge by expressing alarmin and tissue-remodeling genes, highlighting significant functional heterogeneity [40]. These keratinocyte subpopulations were shown to be involved in crucial molecular activities, including transcriptional programming, intercellular communication, inflammatory regulation, and modulation of the Wnt signaling pathway. While the mechanisms underlying such compartmentalization remain unclear, they may involve distinct differentiation trajectories or epigenetic regulation [41]. Using scRNA-Seq, we investigated the cellular heterogeneity of the epidermis following *Supt6* knockout and identified distinct keratinocyte subpopulations that may contribute to both enhanced epidermal differentiation and the initiation of the inflammatory response. Spatial and temporal profiling of these subpopulations will help determine whether the observed proinflammatory responses are intrinsic to a subset of basal keratinocytes or reflect a transient state induced by external stimuli in response to environmental cues [41]. On the basis of these insights, we concur with a previous study in which the epidermis may function in compartmentalization in response to different environmental and genetic cues.

Interestingly, we observed consistent downregulation of Wnt signaling following *Supt6* knockout by both bulk RNA-Seq and enrichment of basal keratinocyte subpopulations by scRNA-Seq, which was associated with a significant delay in wound healing and the hair cycle. Given the well-established role of Wnt signaling in skin regeneration, including hair follicle development [12, 42], these findings underscore its importance in SPT6-mediated epidermal repair. Interestingly, our previous in vitro study using differentiated human keratinocytes did not show a similar suppression of Wnt signaling. This discrepancy suggests that the phenotype may be species-specific or context-dependent, potentially because of the high density of hair follicles in mouse skin compared with that in human skin. Additionally, Wnt/β-catenin signaling has been shown to regulate the localization of hemidesmosome components specifically in the basal layer of keratinocytes but not in differentiated keratinocytes [43], supporting a basal layer-specific function for Wnt signaling in epidermal integrity and regeneration.

Our data indicate that SPT6 is essential for suppressing epidermal inflammatory responses and that its disruption may contribute to the pathogenesis of inflammatory skin diseases, such as psoriasis. In support of this, a transcriptomic correlation analysis revealed strong similarity between the skin of *Supt6*-KO mice and that of IMQ-treated mice, with a high correlation coefficient (*r* = 0.85) based on two independent GEO datasets [15, 16]. Furthermore, comparative transcriptome profiling of *Supt6*-KO epidermis and lesional skin from psoriasis patients revealed a significant, moderate positive correlation, further indicating that SPT6 is involved in psoriasis-like pathology. Consistently, the topical application of IMQ to *Supt6*-KO mice exacerbated the inflammatory response, as evidenced by increased erythema and scaling. These findings support the role of SPT6 in suppressing the epidermal inflammatory response and suggest that *Supt6* deficiency sensitizes the skin to inflammatory stimuli, thereby predisposing it to diseases such as psoriasis. Importantly, the phenotype observed in *Supt6*-KO mice is unlikely to result from secondary bacterial, fungal, or viral infection due to epidermal barrier disruption but rather reflects a type of sterile inflammation [44]. This conclusion is supported by the observation that mice treated with a combination of six antibiotics for ten days still exhibited increased epidermal thickness and elevated expression of differentiation markers. On the basis of these findings, we continued to investigate the mechanisms by which SPT6 restrains cell-intrinsic and sterile inflammation in basal keratinocytes.

Abnormal activation of NF-κB has been associated with several inflammatory diseases, such as arthritis and psoriasis [45–48]. However, its role in psoriasis remains controversial, as NF-κB inhibition has been shown to promote keratinocyte proliferation [45]. Notably, NF-κB-driven positive feedback loops are hallmarks of chronic inflammation and cancer [49, 50]. However, given the essential role of NF-κB in numerous physiological processes, global inhibition of this pathway often results in toxicity. Our study presents a potential alternative strategy to dampen inflammation by selectively targeting *RELA* enhancer elements, which may offer higher specificity and lower toxicity. More broadly, this work underscores the importance of enhancer regulation in tuning epithelial immune responses and provides a conceptual framework for understanding transcriptional control in barrier tissue inflammation.

We observed a significant increase in epidermal thickness coupled with enhanced epidermal differentiation. This was evidenced by the marked expression of early and terminal differentiation markers, including KRT1 and LOR. Interestingly, these findings contrast with our previous report that SPT6 promoted epidermal differentiation and stratification in differentiated human keratinocytes [13]. One possible explanation for this discrepancy is that SPT6 may function differently in basal layer keratinocytes than in differentiated keratinocytes. SPT6 likely interacts with different downstream partners, leading to context-dependent effects. Alternatively, the observed differences could stem from variations between the in vivo and in vitro settings used to investigate SPT6 function, highlighting the importance of physiological microenvironments in modulating SPT6 function. Finally, it could be a consequence of chronic inflammation. In support of this, pseudotemporal trajectory analysis revealed that the expression of genes associated with increased epithelial cell differentiation was upregulated in *Supt6*-KO mice in parallel with the activation of inflammatory signaling pathways, including cytokine‒cytokine receptor interactions, JAK–STAT signaling, and TNF signaling, which are known to play central roles in the pathogenesis and development of skin inflammatory diseases, such as psoriasis [18, 51]. Together, these findings suggest that SPT6 plays a multifaceted, context-dependent role in maintaining epidermal homeostasis.

Our cell–cell communication analysis revealed that specific pathways were altered in *Supt6*-KO epidermis. Eph-ephrin signaling is known to regulate key processes, such as developmental processes, cell adhesion, motility, survival, proliferation, and differentiation. Previous studies have demonstrated that EFNA signaling can promote epidermal differentiation while suppressing genes involved in cell adhesion, wound healing-associated signaling, and receptor- and ECM-associated genes. Although promiscuous, Efn-Eph interactions in the epidermis are not functionally redundant and are context specific [52]. The Eph/ephrin axis has been associated with cutaneous malignancy, wound healing defects, and inflammatory skin conditions [53]. Notably, EphA2/ephrin-A1 signaling is activated in psoriasis, with downstream effects on abnormal epidermal differentiation indicated by the induction of *S100A7* [54], a marker of psoriatic lesions. In addition to changes in Eph-Efn signaling, interactions between *App* and *TNFRSF21* were significantly decreased in the *Supt6*-KO epidermis, particularly in subclusters 4 (Interm-3) and 5 (Bas-2). *TNFRSF21* is known to be expressed on endothelial cells and has been implicated in tumor cell-induced necroptosis, facilitating tissue extravasation and metastasis [55]. The reduction in *App*-*TNFRSF21* interaction may reflect disrupted epidermal homeostasis and impaired communication networks critical for maintaining skin integrity.

Although the *Supt6*-KO epidermis exhibits enhanced terminal differentiation at both the phenotypic and marker levels, trajectory and differentiation-potential analyses revealed that this differentiation is not accompanied by orderly lineage progression. CytoTRACE analysis revealed reduced transcriptional plasticity in *Supt6*-KO keratinocytes, which is consistent with premature commitment to differentiated states. In parallel, Monocle2 and Slingshot analyses demonstrated disrupted directional flow along differentiation trajectories, with aberrant accumulation of cells in basal and intermediate states. Together, these findings support a model in which SPT6 loss drives accelerated or aberrant terminal differentiation while impairing coordinated progression through epidermal differentiation trajectories. Consequently, the enhanced terminal differentiation observed in the *Supt6*-KO epidermis likely reflects an inflammation- or stress-associated differentiation program rather than normal homeostatic differentiation. We propose that SPT6 is essential for maintaining epidermal homeostasis because it preserves transcriptional plasticity and ensures orderly lineage differentiation, and that its loss causes premature terminal differentiation coupled with disrupted differentiation dynamics and inflammatory activation.

The skin, as a frontline barrier that is continuously exposed to microbial, mechanical, and UV challenges, maintains a state of low-threshold readiness to respond rapidly to inflammatory signals. This poised state is supported by several intrinsic features. First, keratinocytes express a broad array of pattern recognition receptors (PRRs), including Toll-like receptors (TLRs), RIG-1-like receptors (RLRs), NOD-like receptors (NLRs), and the cGAS-STING pathway, which enables rapid detection of microbes or cells. These PRRs recognize danger-associated molecular patterns (DAMPs) and pathogen-associated molecular patterns (PAMPs), thereby activating downstream inflammatory pathways. Second, basal keratinocytes maintain accessible chromatin at enhancers for NF-κB and AP-1 target genes [56, 57], facilitating rapid induction of proinflammatory responses. While these features enable the epidermis to respond swiftly to environmental or cellular insults, they also confer latent susceptibility to spontaneous or exaggerated inflammatory activation.

Within this primed context, autoinflammation can arise not from infection but from failure of intrinsic suppressive mechanisms that normally keep inflammatory gene programs below the activation threshold. Our data demonstrated that SPT6 directly binds to the *RELA* enhancer and blocks a positive transcriptional feedback loop that otherwise amplifies NF-κB signaling. The loss of SPT6 results in the release of this brake, leading to the aberrant activation of inflammatory transcriptional programs even in the absence of external threats. Our findings support a model in which active suppression by factors such as SPT6 is required to maintain epidermal homeostasis.

Other intrinsic suppressive mechanisms have been identified that support this paradigm. For example, ZNF750, which is highly expressed in differentiated keratinocytes, dampens immune responses by inhibiting PRR expression [10], whereas TSLP-mediated alarmins released by barrier epithelium can be regulated through the TSLP/ILC axis [58]. The requirement for multiple independent transcriptional regulators to restrain basal inflammatory tone suggests that epidermal homeostasis depends on coordinated suppressive networks rather than single checkpoints. Together, these findings establish that the epidermis exists in a primed inflammatory state and that disruption of key transcriptional suppressors such as SPT6 is sufficient to trigger autoinflammation.

We acknowledge that this study is limited by the lack of analysis on interactions between epidermal and dermal cells in the scRNA-Seq dataset. Our current investigation focused specifically on dissecting the epidermal subpopulation following *Supt6* knockout. Future studies will explore epidermal-dermal cell communication in greater detail to gain a more comprehensive understanding of tissue-level responses. While our differential gene expression, SCENIC regulon analysis, and target gene validation provide strong evidence for transcriptional alterations, direct functional validation through approaches such as CRISPR perturbation of identified regulons or spatial transcriptomics would further strengthen these findings and represent an important direction for future studies.

## Materials and methods

### Conditional *Supt6* knockout mouse model

All animal studies were conducted in accordance with the Declaration of Helsinki principles, with approval from an Institutional Review Board protocol approved by The First Affiliated Hospital of Sun Yat-sen University (reference no. 2024-141). The mice were housed under specific-pathogen-free (SPF) conditions at the animal facility of The First Affiliated Hospital of Sun Yat-sen University. All the mice were maintained in the same room under standardized conditions: a constant temperature of 21 °C, 45–65% humidity, and a 12-h light‒dark cycle, with ad libitum access to food and water.

Conditional *Supt6* knockout (*Supt6*-KO) mice (K14-CreERT; *Supt6*^flox/flox^) were generated in-house by crossing *Supt6*^flox/flox^ mice (generated by Biocytogen) with a transgenic Keratin 14 (K14)-CreERT-expressing mouse, which was kindly provided by Dr. Demeng Chen (The First Affiliated Hospital of Sun Yat-sen University). Homozygous mice were used for breeding and experimentation. Genotyping and tissue-specific deletion were performed by PCR, and SPT6 deletion in skin was confirmed by both western blotting and real-time quantitative PCR (RT‒qPCR).

To activate Cre recombinase and induce SPT6 deletion, tamoxifen (MedChemExpress: HY-13757A) was dissolved in corn oil (MACKLIN: C805618) to a final concentration of 50 mg/kg and administered *via* intraperitoneal injection once daily for five consecutive days, with the control group receiving the same dose of corn oil.

### Genotyping

The genotypes of K14-CreERT and *Supt6*^flox/flox^ mice were determined by PCR using tail-derived genomic DNA. Specific primer pairs were designed to distinguish wild-type (WT), floxed, and Cre-recombined alleles. *Supt6*^flox/flox^ mice: Primer pair 1 (F: 5′-CTTTGCAGAATTTGCAGCATAGGTC-3′; R: 5′-ATTGCCTGATACCTTTCCTGTTTGC-3′) amplifies a 484-bp product in WT mice and a 618-bp product in *Supt6*^flox/flox^ mice. Primer pair 2 (F: 5′-CTGGCCTTGAACTCGCAGAGATTC-3′; R: 5′-AAAGAGGTGATGACGGGGAGAAG-3′) generates a 318-bp amplicon in WT mice and a 403-bp product in floxed mice. K14-CreERT mice: Cre recombinase expression was verified using primers (F: 5′-CCATCTGCCACCAGCCAG-3′; R: 5′-TCGCCATCTTCCAGCAGG-3′), yielding a 250-bp product in K14-CreERT-positive mice. No amplification was observed in the WT mice. *Supt6* DNA recombination analysis: Cre-mediated recombination of the *Supt6*-flox allele was confirmed with primers (F: 5′-CTTTGCAGAATTTGCAGCATAGGTC-3′; R: 5′-AAAGAGGTGATGACGGGGAGAAG-3′). Amplification produced a 4868-bp fragment in WT mice, whereas postrecombination (Mut) alleles yielded a shorter 457-bp product. PCRs were performed using a thermal cycler with the following cycling parameters: initial denaturation (95 °C for 3 min), 40 cycles of (i) denaturation (94 °C for 25 s), (ii) annealing (62 °C for 25 s), (iii) elongation (72 °C for 15 s), and a final extension (72 °C for 5 min). PCR products were resolved on a standard 2% agarose gel (Biosharp: BS081) and visualized using standard gel electrophoresis methods.

### In vivo mouse skin wound healing

*Supt6*-KO and control mice, aged 6 weeks, were used for the wound healing experiments. Following the final tamoxifen injection, one 6-mm-diameter full-thickness excisional incision was created on the dorsal skin of each mouse using a sterile biopsy punch. Wounds were left to heal by secondary intention without suturing. Wound areas were photographed and measured by ImageJ at 0, 2, 4, and 7 days post-wounding to assess healing progression. The quantified wound area is expressed as a percentage of the initial wound size on day 0.

### Systemic treatment of mice with an antibiotic cocktail

*Supt6*-KO mice that received intraperitoneal injections of tamoxifen at 6 weeks of age to activate *Cre* were divided into two groups. To suppress the microbial flora, the mice were administered a broad-spectrum antibiotic cocktail (0.4 mg/mL kanamycin, 0.035 mg/mL gentamycin, 0.057 mg/mL colistin, 0.215 mg/mL metronidazole, 0.045 mg/mL vancomycin, and 0.01 mg/mL erythromycin) in 1% sucrose-containing drinking water, starting from the first day of tamoxifen injection and continuing until tissue harvest. The other group received only 1% sucrose in their drinking water.

Fresh antibiotic- or sucrose-containing water was prepared and replenished every three days to maintain stability and effectiveness. Phenotypic evaluations, including body weight monitoring and histological analysis (H&E staining) of dorsal skin and ear tissue, were conducted at 5 and 10 days after the final tamoxifen injection. To verify the efficacy of microbial depletion, fecal samples were collected, homogenized, and plated on LB agar plates without antibiotics. The bacterial colony density was quantified to assess microbial suppression.

### Detection of fungi and viruses on mouse skin surfaces

Dorsal skin samples from control and *Supt6*-KO mice were collected on day 10 after the final tamoxifen injection, fixed in 10% paraformaldehyde, and embedded in paraffin. Periodic acid–Schiff (PAS) staining was performed to detect fungal components. Briefly, the paraffin sections were deparaffinized, rehydrated, oxidized with periodic acid, treated with Schiff reagent, counterstained with hematoxylin, and mounted for microscopic observation. To culture fungi from the skin surface, sterile cotton swabs premistened with sterile PBS were used to rub the dorsal skin of control and *Supt6* KO mice 20 times. The swabs were rinsed in sterile PBS, and the suspension was plated onto Sabouraud dextrose agar (SDA) and potato dextrose agar (PDA) plates supplemented with gentamicin (40 mg/L) and chloramphenicol (50 mg/L) to suppress bacterial growth. The plates were incubated at 30 °C for 3 days. Fungal colonies were imaged and quantified. The dorsal skin surfaces of the mice were also gently scraped with a sterile surgical blade. Skin scrapings were immediately placed into fungal lysis buffer. Fungal DNA was extracted using a fungal DNA extraction kit (Omega Biotek: D3390) according to the manufacturer’s instructions. Equal amounts of DNA were used for quantitative PCR (qPCR) analysis.

Similarly, on day 10 after tamoxifen injection, sterile PBS-soaked cotton swabs were used to rub the dorsal skin 20 times. The swabs were rinsed in sterile PBS, and viral DNA was extracted using a viral DNA extraction kit (Omega Biotek: D3892). Equal amounts of DNA were used for qPCR analysis.

### Skin microbiota transplantation

Skin microbiota transplantation was initiated on the first day of tamoxifen administration and continued until the end of the experiment. The microbiota was collected by swabbing the dorsal skin with sterile PBS-soaked cotton swabs and then transferred to recipient mice by topical swabbing. *Supt6-*KO mice were randomly divided into two groups: one group received skin microbiota from *Supt6-*KO donors, and the other group received microbiota from control donors. On day 10 after tamoxifen injection, skin samples were collected and subjected to hematoxylin and eosin (H&E) staining to evaluate skin phenotypes.

### Imiquimod-induced psoriasis-like model

*Supt6*-KO mice and control mice received intraperitoneal injections of tamoxifen starting at 6 weeks of age to induce SPT6 deletion, followed by shaving of the dorsal hair. On day 5 after the final tamoxifen injection, 62.5 mg of 5% imiquimod (IMQ) cream (Med-Shine Pharmaceutical, China) was topically applied to a 2 cm × 2 cm shaved area on the dorsal skin of each mouse. The IMQ application was repeated once daily for 3 consecutive days. Skin tissues from the treated area were collected 24 h after the final IMQ application. H&E staining was performed to histologically evaluate psoriasiform phenotypes, including epidermal hyperplasia, immune cell infiltration, and parakeratosis.

### Tissue processing and histology analyses

Mouse tissues were fixed in 10% neutral-buffered formalin at room temperature overnight, transferred to 75% ethanol, and embedded in paraffin. Histologic sections (5 μm) were stained with H&E or processed for immunohistochemistry. Immunohistochemical staining was carried out using an anti-MPO primary antibody (Abcam: ab208670) at 1:1000, following a standard protocol. Microscopy images were captured using a Leica DMI microscope equipped with a Leica DCF450 camera (Leica Microsystems, Wetzlar, Germany) or a Nikon Eclipse 80i (Tokyo, Japan) microscope fitted with a Nikon DS-Qi1Mc camera. Images were processed with Photoshop CS (Adobe, San Jose, CA).

### Transmission electron microscopy

Mouse dorsal skin tissues (1 mm^3^) were promptly dissected after cervical dislocation and fixed in 2.5% glutaraldehyde in 0.1 M phosphate buffer (pH 7.4) at 4 °C for 24 h. Following three phosphate buffer rinses, the samples were postfixed with 1% osmium tetroxide (OsO₄) for 2 h at 4 °C and then dehydrated through an ethanol series (50% to 100%) and acetone. The tissues were then infiltrated with epoxy resin, embedded, and polymerized at 60 °C. Ultrathin sections (70 nm) were cut using an ultramicrotome, double-stained with uranyl acetate and lead citrate, and examined under a Talos L120C transmission electron microscope at 80 kV. Quantitative analysis was performed using ImageJ (NIH, USA).

### Isolation of human epidermal stem and progenitor cells

Primary human epidermal keratinocytes were isolated from discarded foreskin tissues of young children (aged 5–10 years, *n* = 5 donors) undergoing circumcision. Tissue samples were collected with informed consent from patients and parents under a protocol approved by the Institutional Review Board of The First Affiliated Hospital of Sun Yat-sen University (reference no. 2023-216). All the samples were deidentified prior to processing and classified as nonhuman subjects. The study was conducted in accordance with the Declaration of Helsinki principles.

### Cell culture, gene knockdown, poly(I: C) treatment and QNZ treatment

Primary keratinocytes were propagated in Epilife medium (Thermo Fisher Scientific: MEPI500CA) with the manufacturer’s recommended supplements. For gene knockdown experiments, passage 3 or 4 keratinocytes were transfected with 20 nM siRNA targeting SPT6 (a previously verified siRNA sequence against *SUPT6H*: GAGCUGAGCUGUCGAUAUA [13]) or a nontargeting control siRNA using Lipofectamine RNAiMAX (Thermo Fisher Scientific: 13778150) according to the manufacturer’s protocol. The cells were incubated with the transfection reagent for approximately 18 h. After transfection for 3 days, control or SPT6 knockdown cells were incubated with high-molecular-weight poly(I: C) (InvivoGen: tlrl-pic) at 1 μg/mL for four h before harvest. For QNZ treatment, 5 μM QNZ (Selleck: S4902) was added to the cells for 20 h before they were harvested.

### Quantitative real-time PCR

Total RNA from cells or tissue was extracted using TRIzol reagent (Thermo Fisher Scientific: 15596026) according to the manufacturer’s instructions. RNA quality and quantity were analyzed using a Nanodrop. One microgram of total RNA was reverse transcribed into cDNA using the PrimeScript™ RT Reagent Kit (Takara: RR047) on an ABI Veriti 96-Well Thermal Cycler (Applied Biosystems). Quantitative PCR was carried out with TB Green® Premix Ex Taq™ II (Takara: RR820) on an Applied Biosystems™ QuantStudio™ 5 system. Gene expression levels were calculated using the ^ΔΔ^Ct method, with *L32* used as an internal control for normalization.

To detect fungal DNA, qPCR was performed using three primer pairs targeting the internal transcribed spacer (ITS1 and ITS2) regions. *Gapdh* amplification was used as a positive control to ensure the quality of the DNA and the efficiency of the qPCR process. Equal amounts of DNA were subjected to qPCR for all the samples.

For viral DNA, virus-specific primers targeting common murine viruses, including ECTV (ectromelia virus), MPV-1 (mouse parvovirus type 1), mouse papillomavirus (MusPV1/MmuPV1), murine cytomegalovirus (MCMV), and murine gammaherpesvirus 68 (MHV68), were used. *Gapdh* amplification was included as a positive control to verify DNA quality and qPCR performance. The primer sequences are provided in Supplementary Table 3.

### Western blot

Total protein was extracted using RIPA lysis buffer supplemented with protease inhibitors and quantified using a BCA™ Protein Assay Kit. The resolved proteins were transferred onto PVDF membranes (Millipore: IPVH00010). The primary antibodies used included β-actin (Santa Cruz: Sc-47778) at 1:30000, SPT6 (Abcam: ab32820) at 1:3000, NF-κB p65 (Proteintech: 66535-1-Ig) at 1:1000, and phospho-NF-κB p65 (Ser536) (Proteintech: 80379-2-RR) at 1:1000 overnight at 4 °C. Secondary antibodies, including HRP-conjugated anti-mouse IgG (Cell Signaling Technology: 7076S) and anti-rabbit IgG (Cell Signaling Technology: 7074S), were used at 1:2000. Protein detection was performed using the enhanced chemiluminescence method (EpiZyme: SQ202), and images were captured and analyzed with an Amersham ImageQuant 800 (Cytiva: 29399481).

### Enzyme-linked immunosorbent assay (ELISA)

The expression levels of IL-1β, CXCL2, and CCL3 in the supernatant of mouse skin homogenates were measured using commercial mouse ELISA kits (IL-1β: Invitrogen, BMS6002-2; CXCL2: Invitrogen, EMCXCL2; CCL3: Invitrogen, EMCCL3) following the manufacturer’s instructions. Briefly, 10 μL of the supernatant, along with 90 μL of sample diluents, was added to the ELISA plate and incubated for 2.5 h at 37 °C. After five washes with wash buffer, streptavidin-HRP solution (100 μL) was added to each well and incubated for 45 min at room temperature. Following five additional washes, 100 μL of chromogenic substrate was added to each well, and the plate was incubated in the dark for 30 min. Finally, stop solution (50 μL) was added to each well. The absorbance at 450 nm was measured within 15 min by using a microplate reader. Total protein extracted from the tissue samples was quantified using a BCA assay. Relative protein levels were calculated by dividing the amount of each detected cytokine by the total protein extracted from the tissue and then normalized to that of the control group.

### Immunofluorescence staining and analysis

Mouse tissue was embedded in optimal cutting temperature (OCT) compound and sectioned at 8 μm with a cryostat. Tissue sections or cultured cells were fixed in 10% neutral buffered formalin for 11 min, followed by blocking in PBS supplemented with 2.5% normal goat serum, 0.3% Triton X-100, and 2% bovine serum albumin for 30 min. The primary antibodies used were Keratin 1 (Biolegend: 905204) at 1:1000, Keratin 14 (Abcam: ab181595) at 1:5000, Loricrin (Abcam: ab198994) at 1:100, NF-κB p65 (Proteintech: 66535-1-Ig) at 1:100, Cleaved caspase 3 (Cell Signaling Technology: 9664) at 1:400, SOX9 (Bioss: bsm-63031R) at 1:500, and Ki67 (Cell Signaling Technology: 71098 T, Abcam: AB16667) at 1:100. The secondary antibodies used were Alexa 594-conjugated donkey anti-mouse IgG (Invitrogen: A21203), Alexa 488-conjugated goat anti-rabbit IgG (Invitrogen: A11034) and Alexa 594-conjugated donkey anti-rat IgG (Invitrogen: A21209) at 1:500. Nuclei were stained with DAPI Fluoromount-G (Southern Biotech: 0100-20). Images were acquired using an Olympus/BX63 upright fluorescence microscope. Quantitative analysis of fluorescence intensity was performed using ImageJ (NIH, USA).

### ChIP‒qPCR

Five million control and SPT6 knockdown cells treated with poly(I: C) for 4 h were harvested, and 3 μg of antibody was used for each antibody pull-down experiment for ChIP [7, 8, 13]. Cells for p65 pulldown ChIP‒qPCR were fixed in both formaldehyde (1% final concentration; Thermo Fisher Scientific: 28908) and disuccinimidyl glutarate (2 mM final concentration; Thermo Fisher Scientific: 20593). As described previously [13, 25, 59], the cells were treated with Farnham lysis buffer and sheared with a syringe to facilitate lysis. After shearing, the cells were centrifuged and resuspended in SDS lysis buffer and sonicated using a Bioruptor® Plus system (Diagenode). Once the appropriate fragment size was achieved, the lysate was incubated with NF-κB p65 (Proteintech: 80979-1-RR) and rabbit IgG (Millipore: 12-370). Fifty microliters of Dynabeads™ Protein G (Invitrogen: 10004D) was added to each sample and rotated for 4 h at 4 °C. Washes were then carried out to reduce nonspecific binding. The beads were then placed in elution buffer, and the supernatant was isolated. Finally, the DNA was decrosslinked and used for downstream ChIP‒qPCR analysis. The RT‒qPCR results are presented as a percentage of the input. The primers used for ChIP‒qPCR are listed in Supplementary Table 3.

### RNA sequencing and subsequent bioinformatic analysis

RNA was purified from primary keratinocytes and mouse dorsal skin using TRIzol following the manufacturer’s instructions. RNA-Seq was performed using the DNBSEQ platform (BGI, China) with PE100. Approximately 40 million pair-ended reads per sample were obtained. Reads were aligned to the human genome hg19 or mouse genome mm10 using Bowtie2 (v2.3.4.3) with default settings [60]. RSEM (v1.3.1) was used to generate gene expression levels, and pheatmap (v1.0.8) was used to construct a heatmap. The differential expression among samples was calculated using DESeq2 [61]. Analysis of the read count distribution indicated that a threshold of ten reads per gene generally separated expressed genes from unexpressed genes; thus, all genes with fewer than ten reads were excluded from DESeq2 analysis. Gene lists for significantly upregulated or downregulated genes were created using *p* < 0.05 and 2-fold change. Enriched GO terms and KEGG analysis for RNA-Seq-identified differentially expressed gene sets were performed using Enrichr and Metascape [62–65]. The predicted p65 binding motif was retrieved from the JASPAR 2026 database (https://jaspar.elixir.no/) [66] using the matrix model MA0107.1. The gene sets used for GSEA [67] were obtained from the Molecular Signatures Database (MSigDB) (https://www.gsea-msigdb.org/gsea/msigdb/index.jsp). Enrichment analysis for KEGG, GO [68], and hallmark pathways [69] was performed to identify functionally relevant biological processes and signaling pathways.

### Single-cell RNA sequencing

#### Tissue dissociation and cell purification

Following euthanasia, the dorsal skin was aseptically excised, and the subcutaneous tissue was removed using a sterile scalpel. The skin was incubated in 0.3% Dispase II solution (prepared in PBS) at 4 °C overnight to separate the epidermis from the dermis. The epidermal layer was carefully dissected, minced into fine fragments, and digested in 0.05% trypsin (diluted in PBS) at 37 °C for 15 min. Trypsin activity was neutralized by the addition of 0.5% bovine serum albumin (BSA) solution. The cell suspension was filtered through a 40-μm cell strainer to remove debris and centrifuged at 300 × *g* for 10 min at 4 °C. Pelleted cells were washed once and finally resuspended in 0.04% BSA. The cells were stained with 0.4% Trypan blue (Thermo Fisher Scientific: 14190144), and the viability was checked on a Countess® II Automated Cell Counter (Thermo Fisher Scientific). The cell suspension was maintained on ice for subsequent experiments.

#### 10x library preparation and sequencing

Single cells and 10x Chromium beads (with UMIs and cell barcodes) were coencapsulated in GEMs at near-saturation loading. Following cell lysis, polyadenylated RNAs were captured on beads *via* hybridization. Beads from all GEMs were pooled for reverse transcription, generating cDNA molecules tagged at the 5’ end (corresponding to the 3’ end of the transcript) with cell-specific barcodes and UMIs. Beads were then subjected to second-strand cDNA synthesis, adaptor ligation, and universal amplification. All the remaining procedures, including library construction, were performed according to the standard manufacturer’s protocol (Chromium Single-Cell 3ʹ v3.1). The sequencing libraries were quantified using a High-Sensitivity DNA Chip (Agilent) on a Bioanalyzer 2100 and a Qubit High-Sensitivity DNA Assay (Thermo Fisher Scientific). Sequencing was performed on an Illumina NovaSeq Xplus platform in PE150 mode.

#### Single-cell RNA-Seq data processing

Reads were processed using the Cell Ranger (v7.1.0) pipeline with default and recommended parameters. FASTQs generated from the Illumina sequencing output were aligned to the mouse genome (version GRCm39) using the STAR algorithm [70]. Gene-barcode matrices were generated for each sample by counting UMIs and filtering non-cell-associated barcodes. The resulting matrix, containing barcoded cells and gene expression counts, was imported into the Seurat R toolkit (v4.1.1) for quality control and downstream clustering analysis [71]. Cells were clustered using graph-based principal component analysis (PCA) followed by modularity optimization. Cell types were annotated using SingleR and validated against established marker gene expression. We visualized the clusters on a 2D map produced with t-distributed stochastic neighbor embedding (t-SNE) [72].

#### Cell–cell communication analysis

CellPhoneDB software (version 4.1.0) [73] (www.cellphonedb.org) was used to explore cell–cell interactions between different cell types. Briefly, for each gene in each cell type, the average expression value of the gene and the percentage of cells expressing the gene were calculated. Potential receptor‒ligand interactions between cell types were inferred on the basis of the expression of receptors in one cell type and ligands in the other, and then the cell type labels of all cells were randomly permuted 1000 times to test the statistical significance of the estimated receptor‒ligand interaction. The intensity of the receptor‒ligand interactions was assessed on the basis of the expression of the ligand‒receptor pairs in the two cell types.

#### Cell cycle analysis

Cell cycle analysis was performed using the CellCycleScoring function implemented in Seurat. On the basis of known sets of cell cycle-related genes, each cell was assigned scores in the G1/S and G2/M phases and classified into the G1 phase, S phase, or G2/M phase.

#### Pseudotemporal dynamics analysis

Pseudotemporal dynamics analysis was performed using the scVelo package [74]. Gene expression dynamics were subsequently modeled as a smooth function of pseudotime using generalized additive models with a negative binomial or quasipoisson family, adjusting for library size and batch. Genes with a significant association with pseudotime were identified using Wald/likelihood-ratio tests (FDR < 0.05, Benjamini–Hochberg). Significant genes were clustered into coexpression modules by hierarchical clustering of smoothed expression profiles or by nonnegative matrix factorization. The heatmaps display the z-scored expression of significant genes ordered by pseudotime and grouped by clusters. The corresponding smoothed expression curve plots were generated on the basis of the average gene expression of each cluster. Functional enrichment for the GO biological process and KEGG pathway terms was performed with g:Profiler/clusterProfiler, using the expressed gene universe and a false discovery rate (FDR) < 0.05.

#### SCENIC

Transcription factor (TF) activity analysis was performed using the “SCENIC” R package, which can infer coexpression regulatory networks between TFs and candidate target genes from the scRNA-Seq dataset [75]. The input matrix was the normalized feature-barcode expression matrix. Briefly, the workflow had three steps: (1) the “GENIE3” R package, which identified potential TF targets on the basis of coexpression; (2) the “RcisTarget” R package, which performed TFmotif enrichment analysis and identified direct targets; and (3) the “AUCell” R package, which scored the activity of the direct targets on single cells.

#### Monocle2

The R package monocle2 (v2.22.0) was used to conduct the pseudotime series analysis. Pseudotime smoothing was implemented via the smoothData() function using default parameters. In brief, DEGs across cell types were initially selected. Next, dimensionality reduction was applied, followed by the construction of a minimum spanning tree. Single-cell expression data were then projected into a low-dimensional space, and the optimal cell ordering was identified. Ultimately, the best-fit developmental or differentiation pseudotime trajectory was established. Briefly, a differential expression analysis was performed to identify the top significantly differentially expressed genes (FDR < 0. 05) between the control and *Supt6* KO conditions to construct the trajectory, and then every single cell was assigned a numeric pseudotime value and then ordered along the trajectory.

#### Slingshot pseudotime trajectory analysis

The R package Slingshot v2.6.094 was used to perform pseudotrajectory analysis of keratinocytes. Default parameters were used, and UMAP input was derived from batch-corrected RNA matrix values generated by Seurat v4 (4.1.1). The trajectory overlay was mapped on cells clustered and annotated by Seurat v4 (4.1.1).

#### CytoTRACE

CytoTRACE [76], a computational tool, was used to obtain a developmental stemness score for each cell, with cells with a high CytoTRACE score being more stem-like and those with a low CytoTRACE score being less stem-like. This computational pipeline involved (i) quantifying gene counts per cell as a differentiation potential indicator, where higher expressed gene numbers correlate with stemness, and (ii) trajectory reconstruction through unsupervised ranking-based dimensionality reduction, mapping cellular progression from least differentiated (high scores) to terminally differentiated states (low scores).

### ChIP-sequencing data analysis

The H3K27ac and H3K4me1 ChIP-Seq datasets generated from proliferating keratinocytes were downloaded from the ENCODE portal [77, 78] (https://www.encodeproject.org/) with the following identifiers: ENCFF757GRD.bigWig and ENCFF142VPB.bigwig. P65 ChIP-Seq data generated in KB cells, an epithelial cell line, were downloaded from GSE52469 [37]. H3K4me1 ChIP-Seq data from proliferating keratinocytes were generated by Dr. Joseph Costello’s laboratory at UCSF. H3K27ac ChIP-Seq data from proliferating keratinocytes were generated by Dr. Bradley Bernstein’s laboratory at Broad.

### Statistical analysis

The results are expressed as the mean ± standard deviation (SD), unless stated otherwise. Statistical comparisons between two groups were performed using an unpaired two-tailed Student’s *t* test. Comparisons among the three groups were performed using one-way ANOVA followed by Tukey’s multiple comparison test. A probability (*p*) value of <0.05 was considered to indicate statistical significance.

## Supplementary information

Supplementary Figures 1-8

Supplementary Table 1

Supplementary Table 2

Supplementary Table 3

Unprocessed WB images

Supplementary Information

## Data Availability

The datasets generated from this study, including RNA-Seq and single-cell RNA-Seq data, have been deposited in GEO (accession numbers: GSE287897 and GSE294435) and the China Genomics Data Center with GSA (HRA010162).
