# Supplementary figures and images for: SPT6 maintains epidermal homeostasis by inhibiting an NF-κB-positive feedback loop to prevent excessive inflammation
Source: Cell Mol Immunol. 2026 Apr 1;23(5):471–90. doi: 10.1038/s41423-026-01410-1 (PMC13129108; doi:10.1038/s41423-026-01410-1)

A

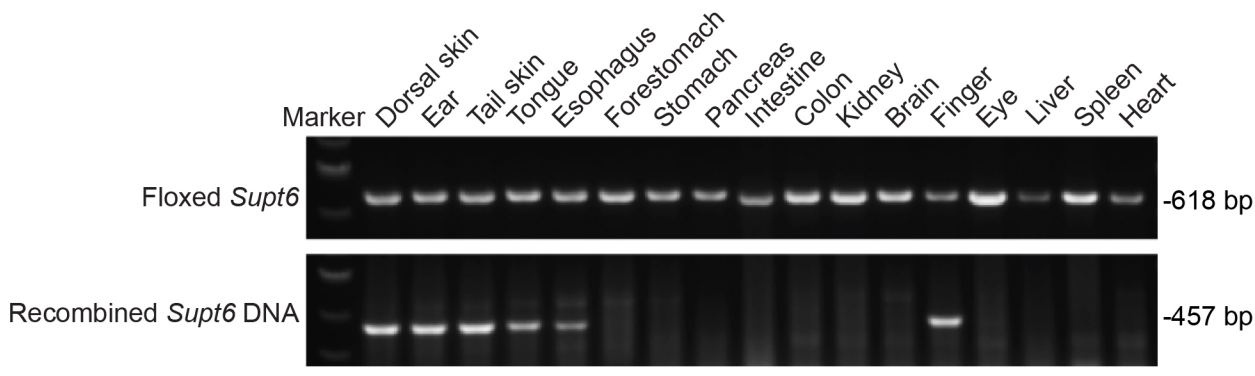

B

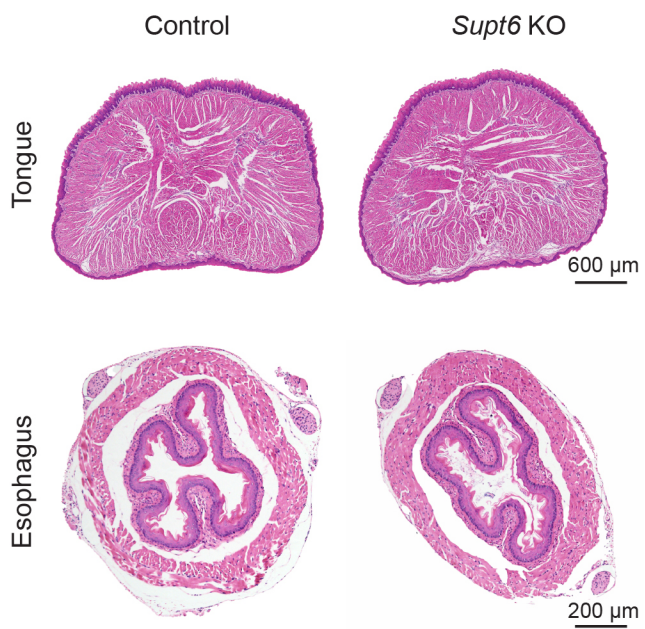

C

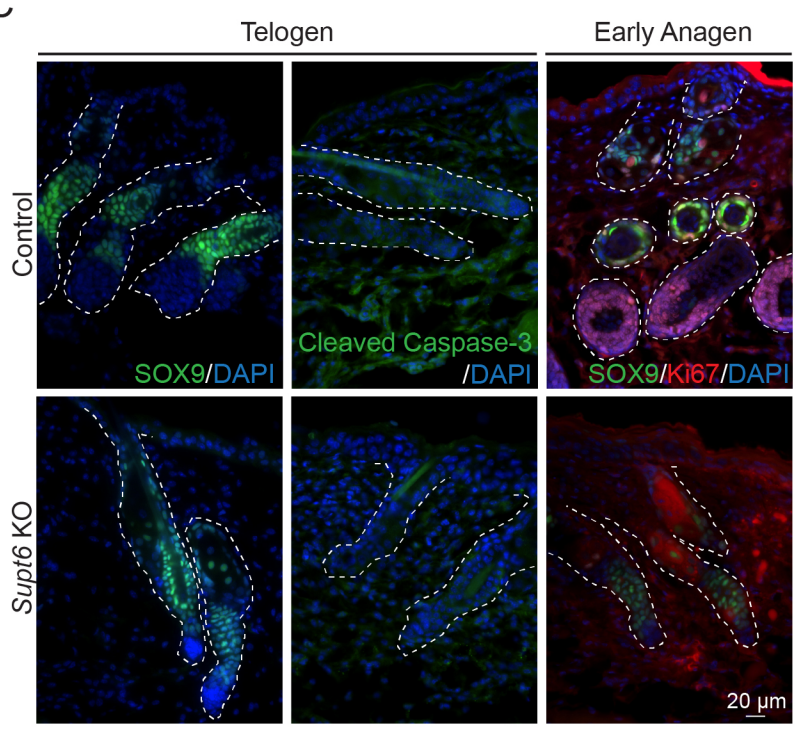

D

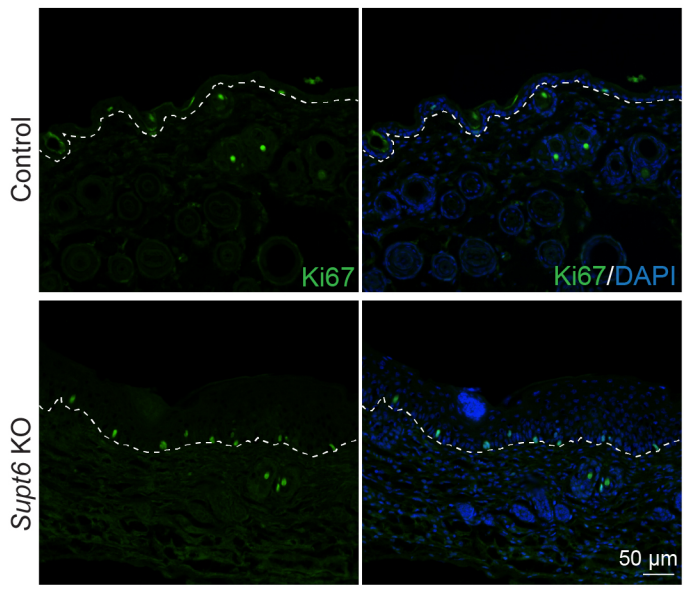

E

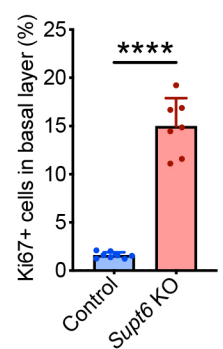

F

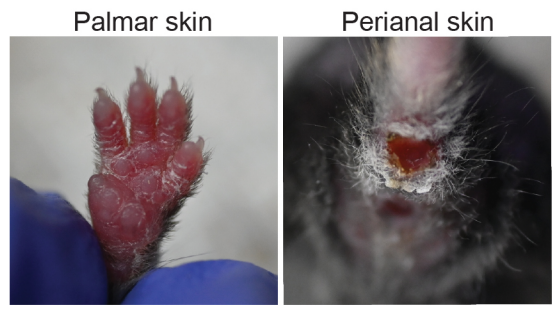

G

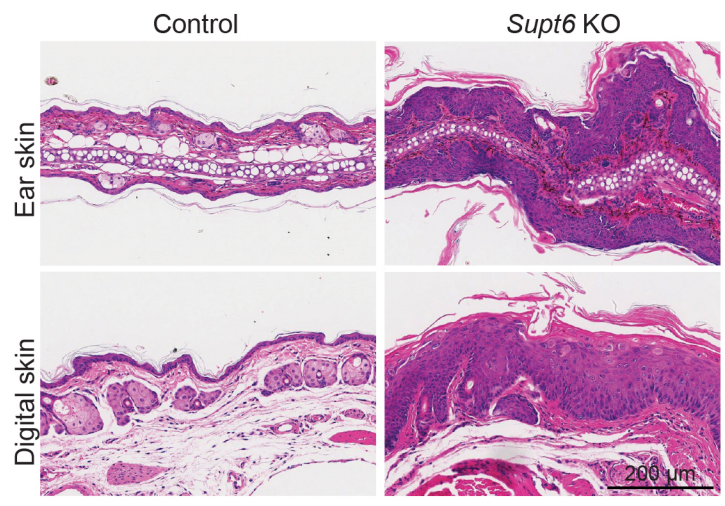

A

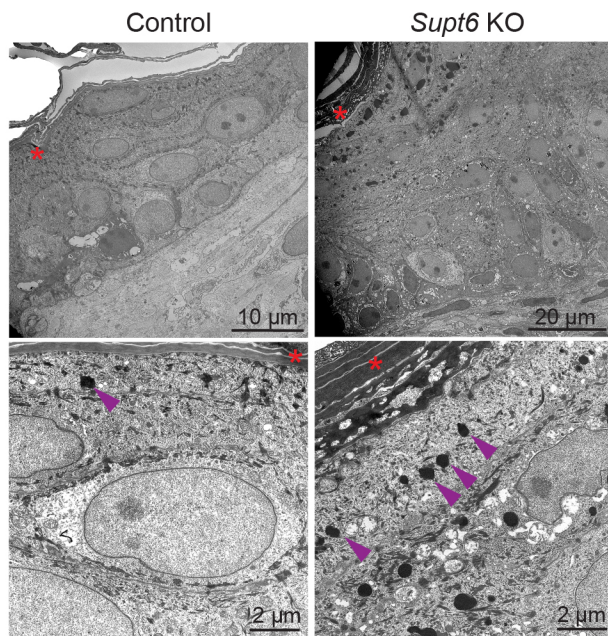

B

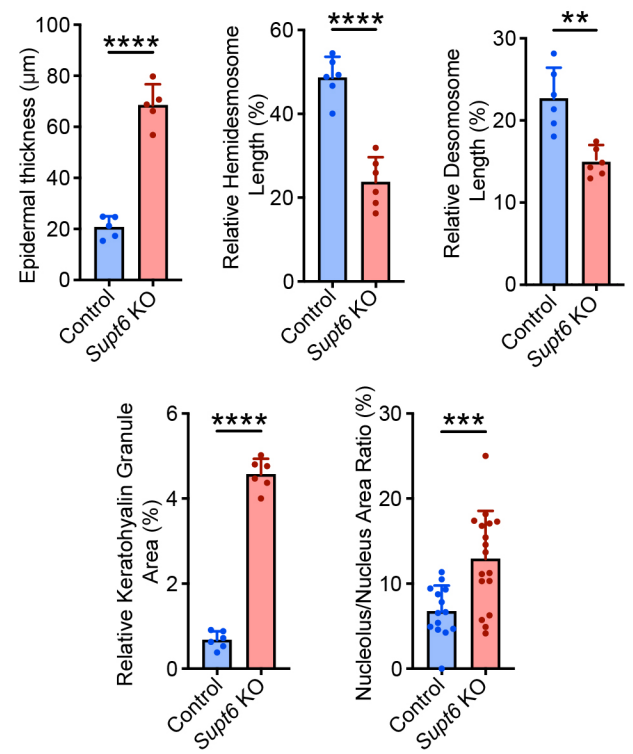

C

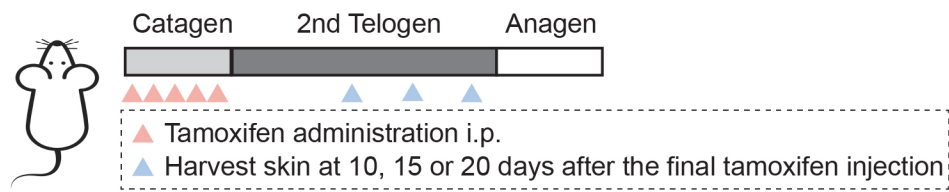

D

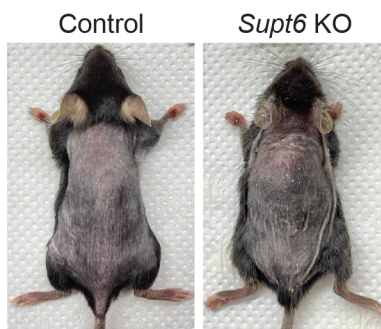

E

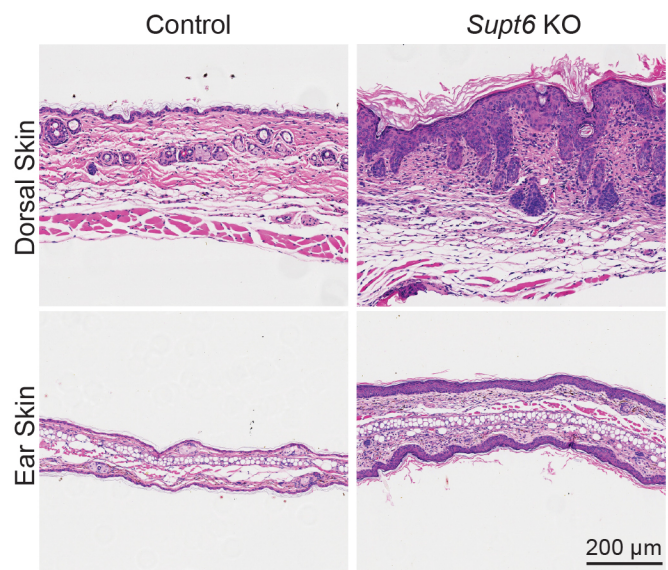

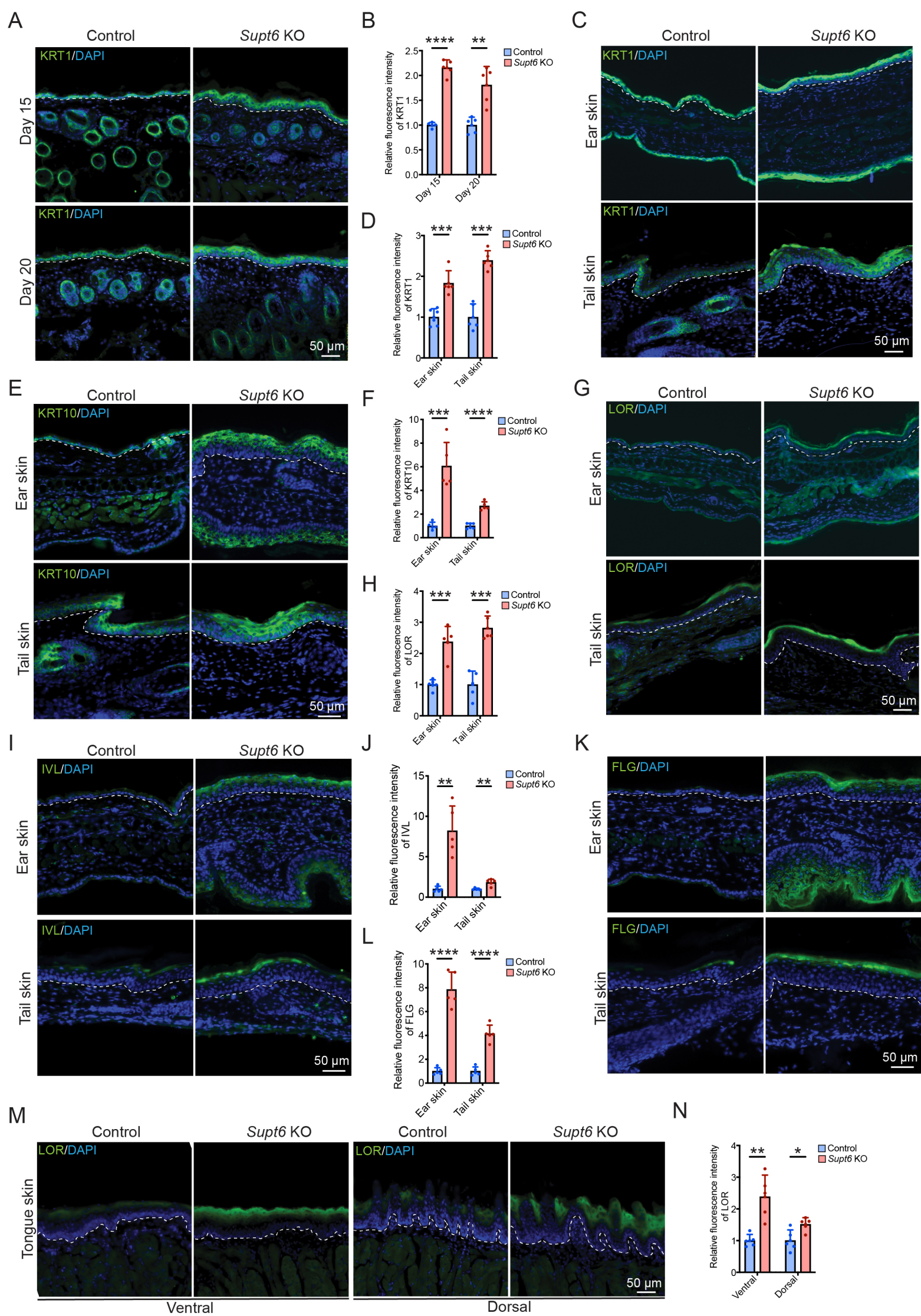

A

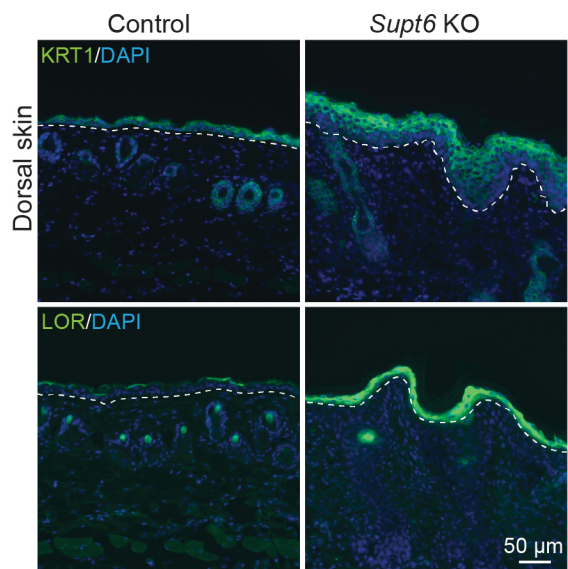

B

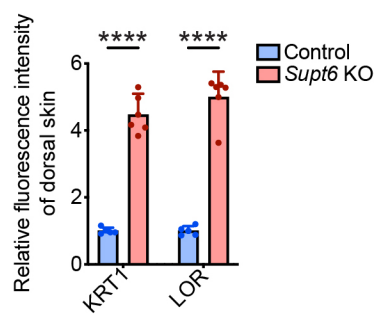

C

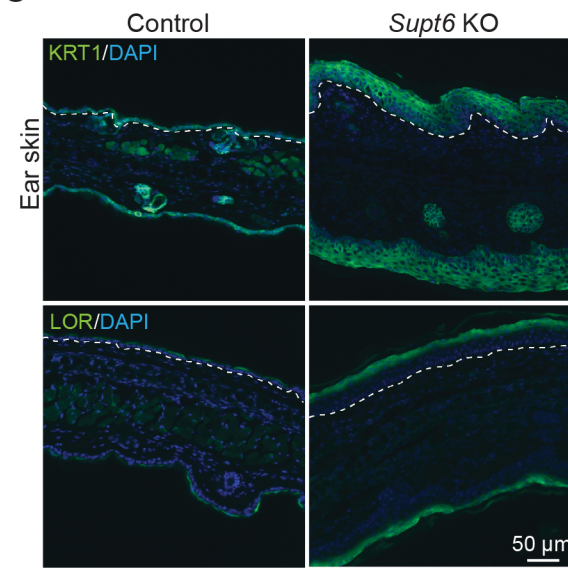

D

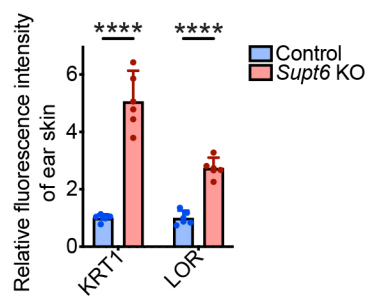

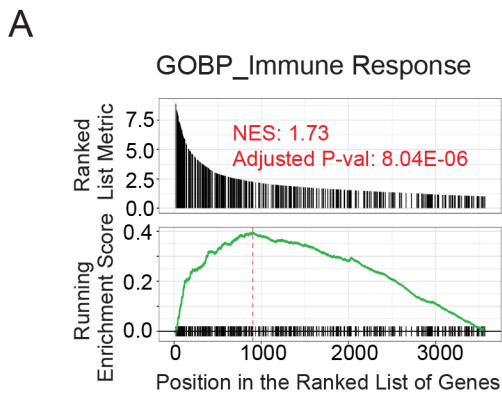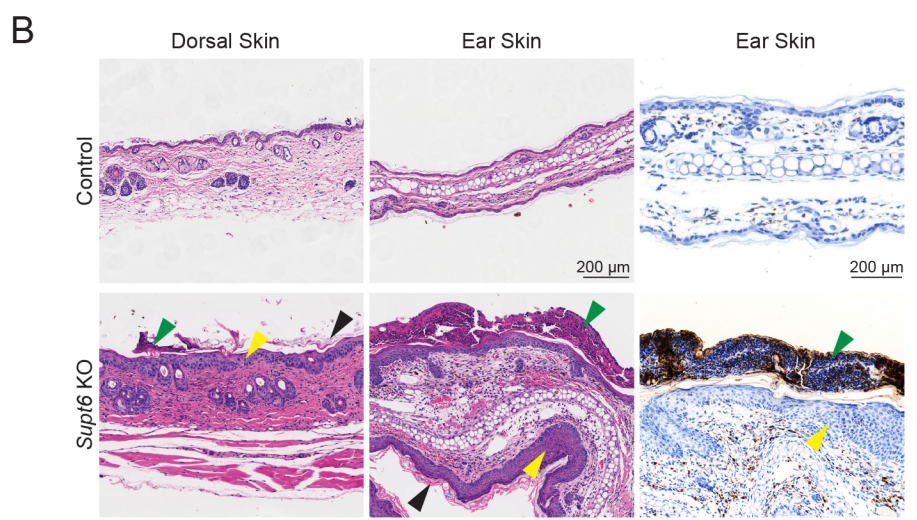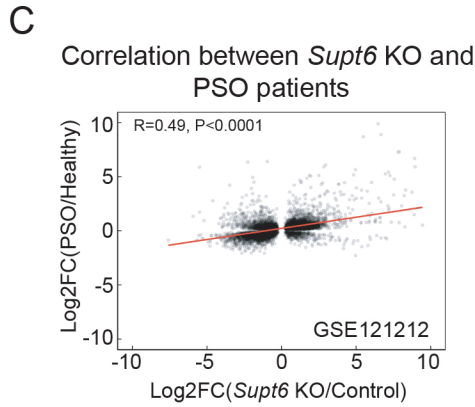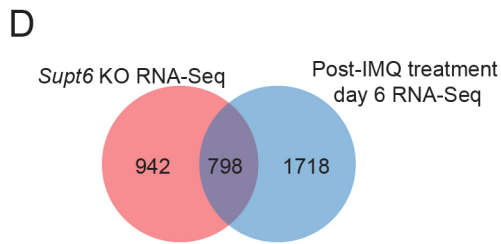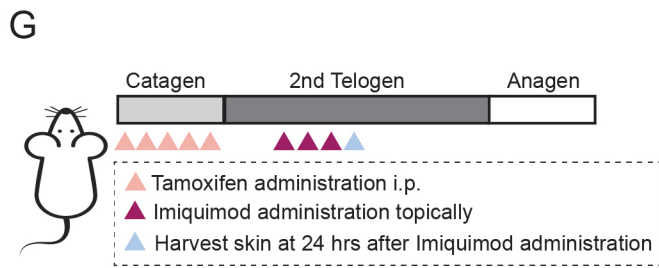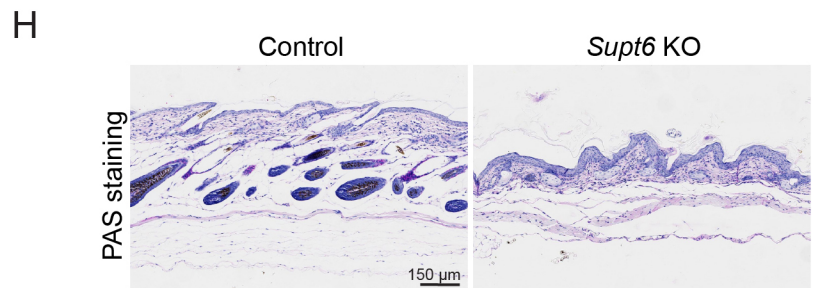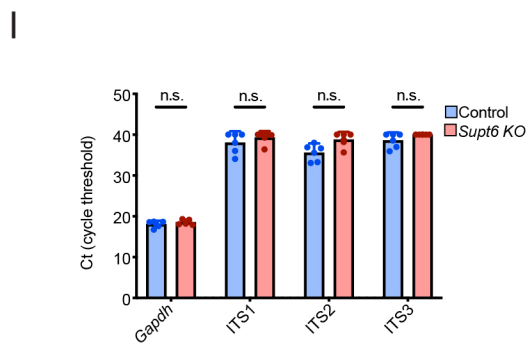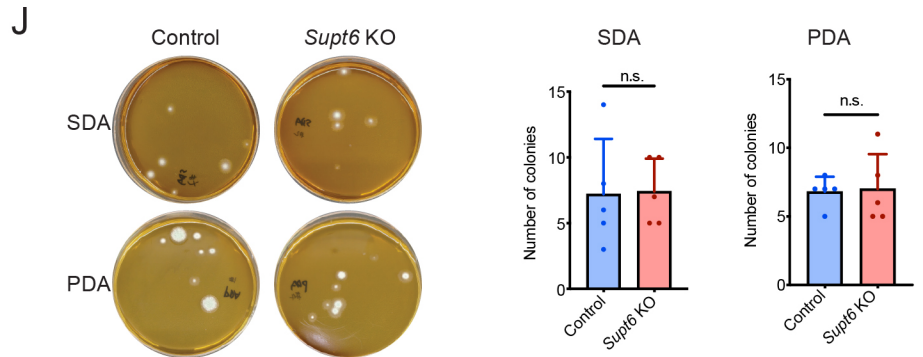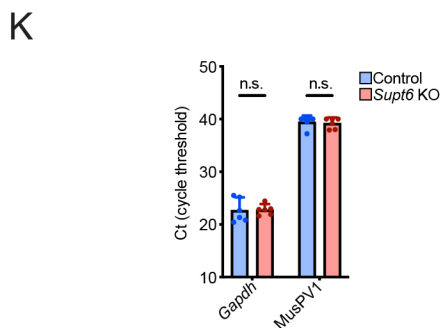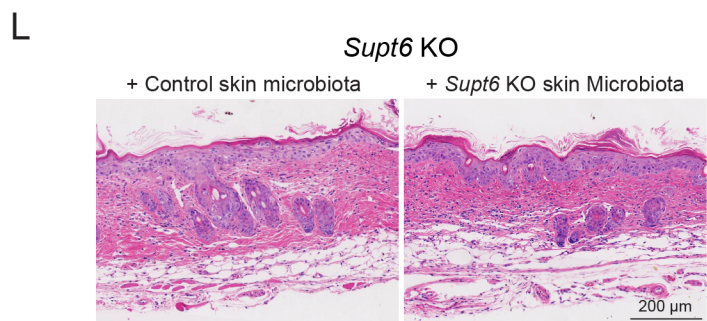

A

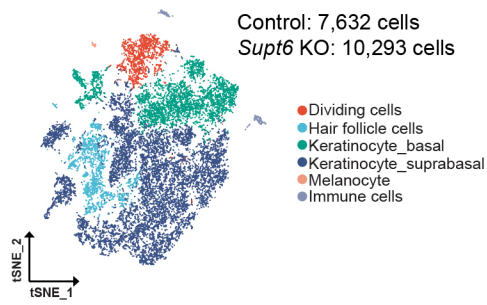

B

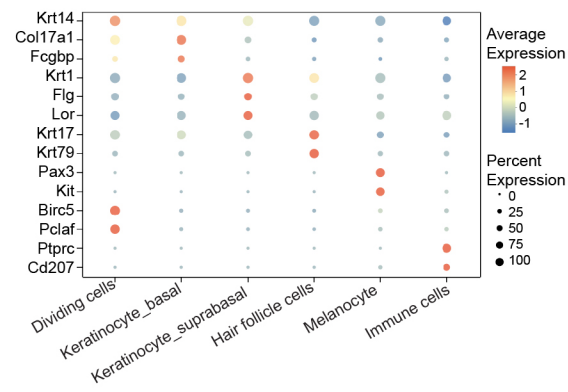

C

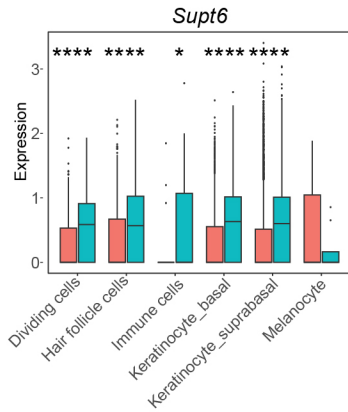

D

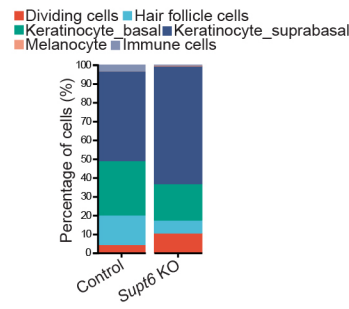

E

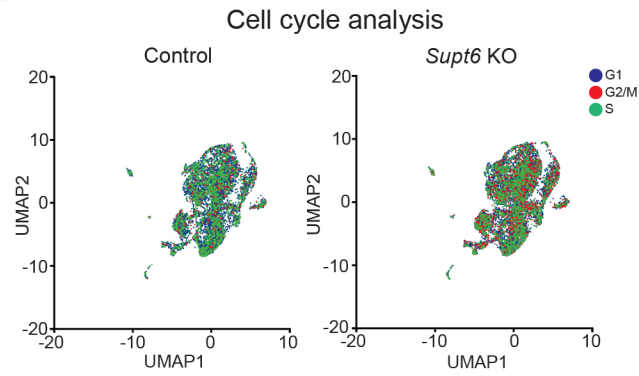

F

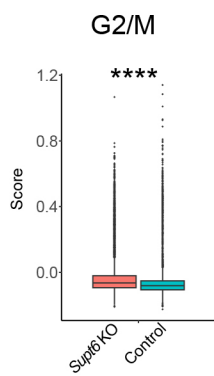

G

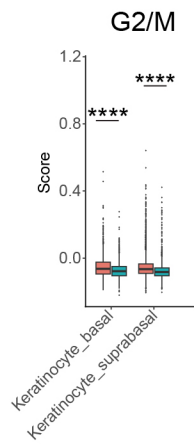

H

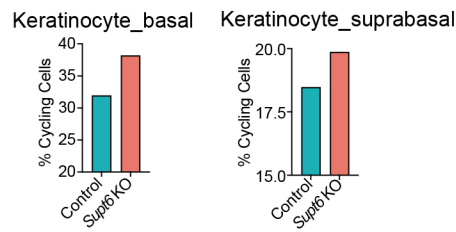

I

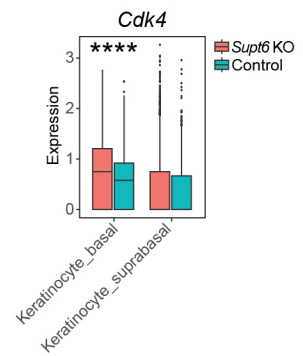

J

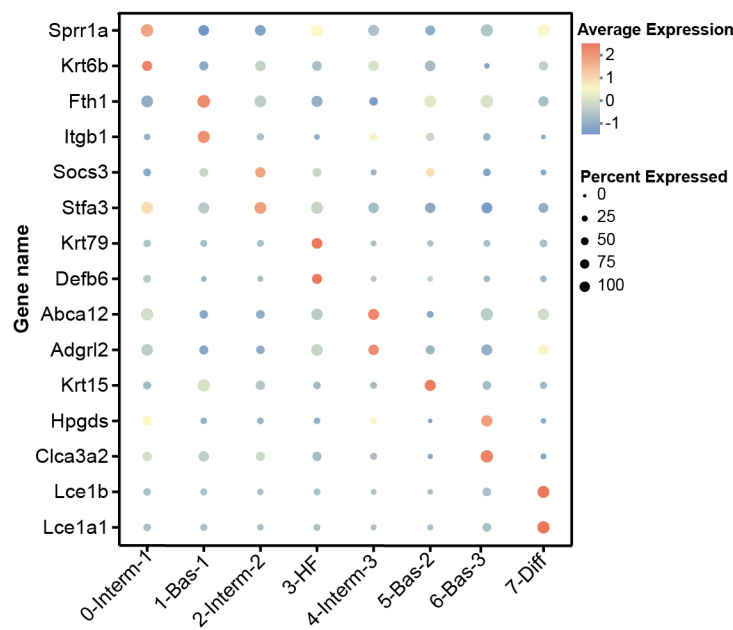

K

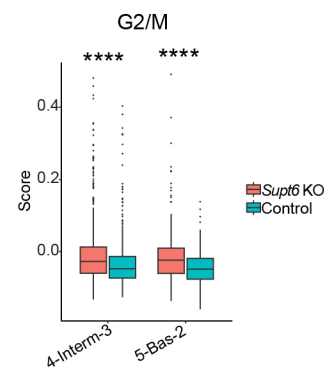

A

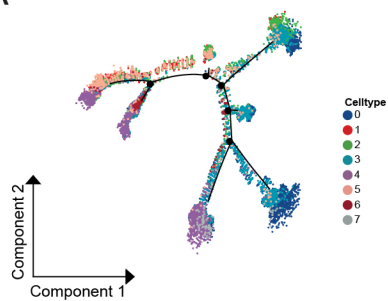

B

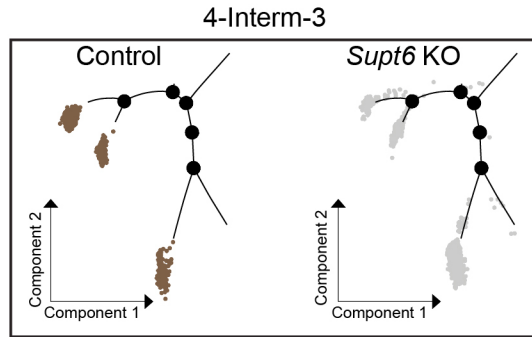

C

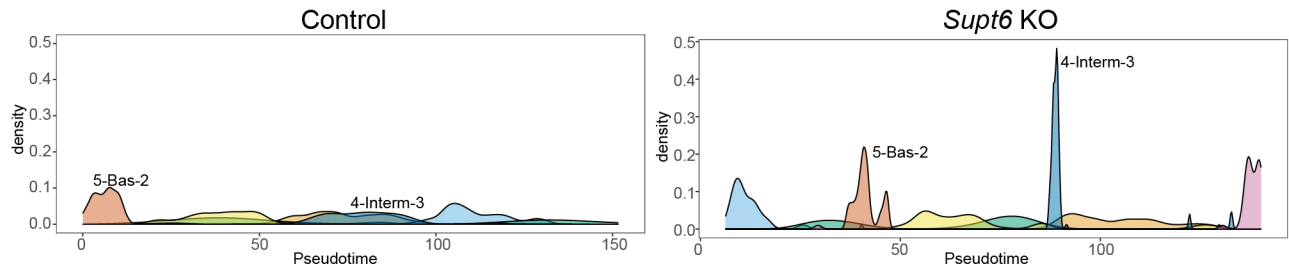

D

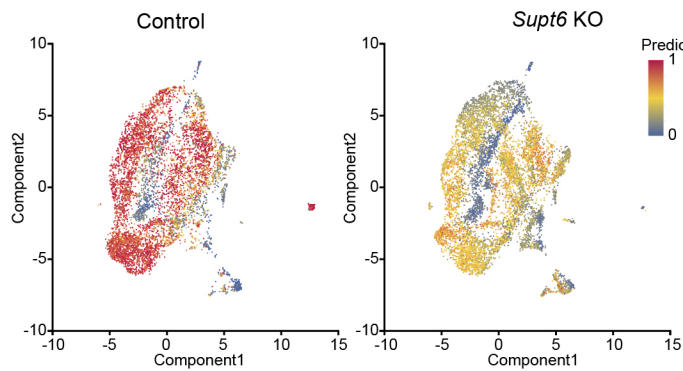

E

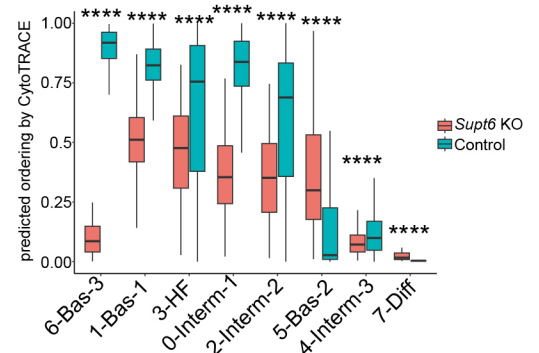

F

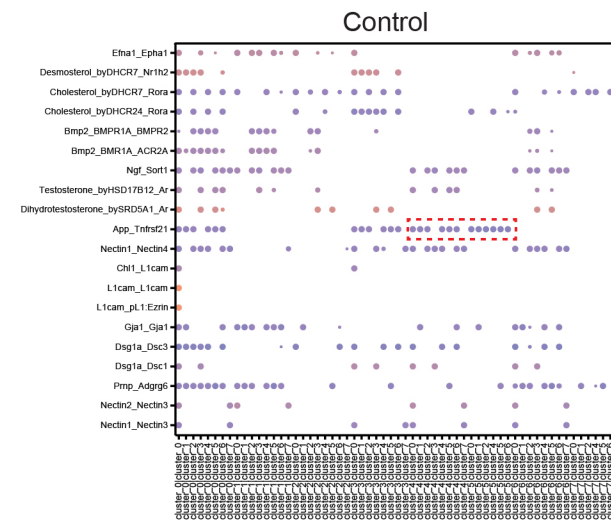

G

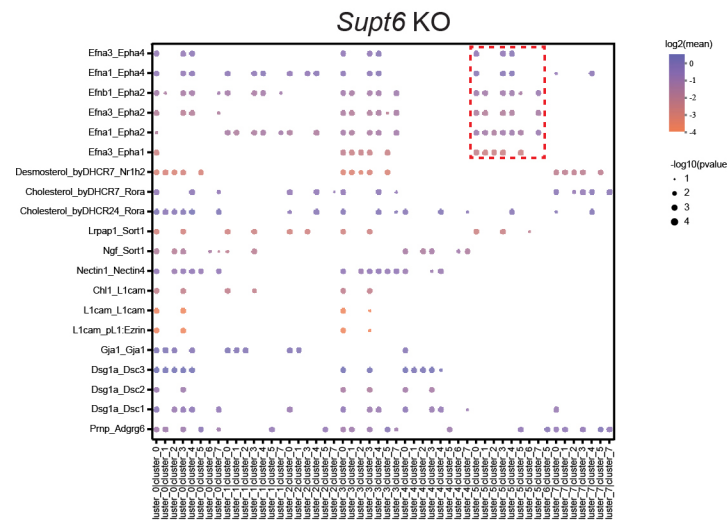

H

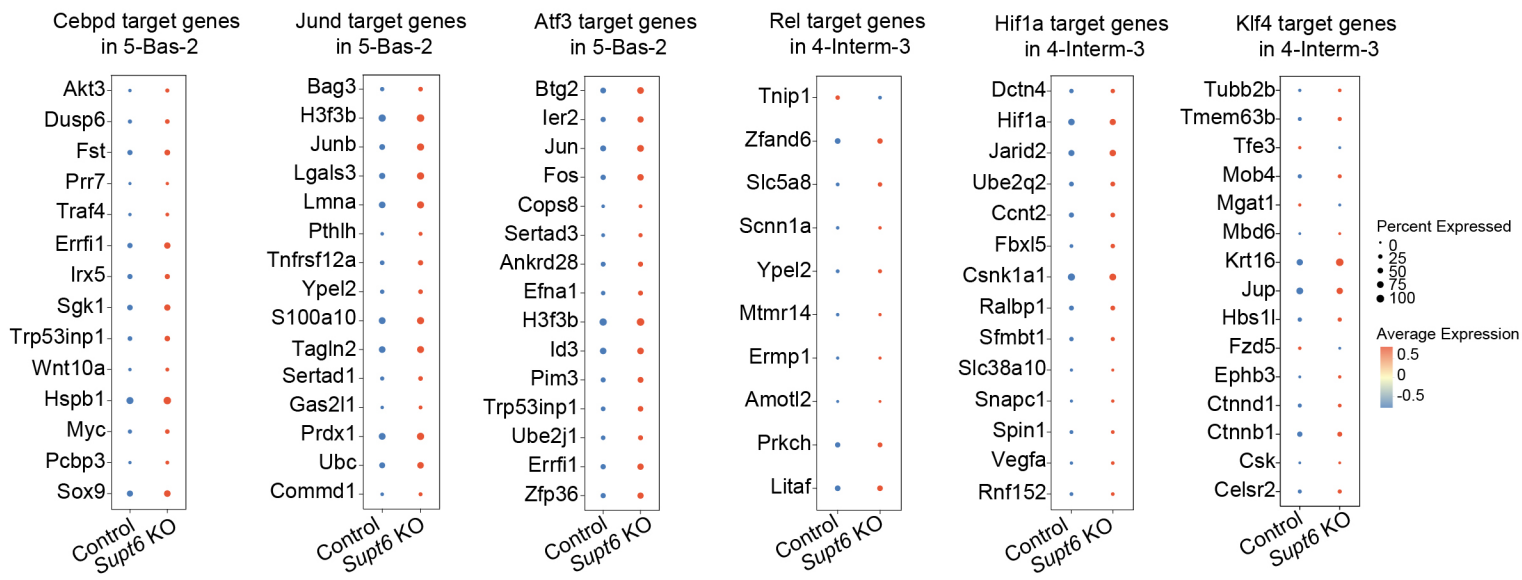

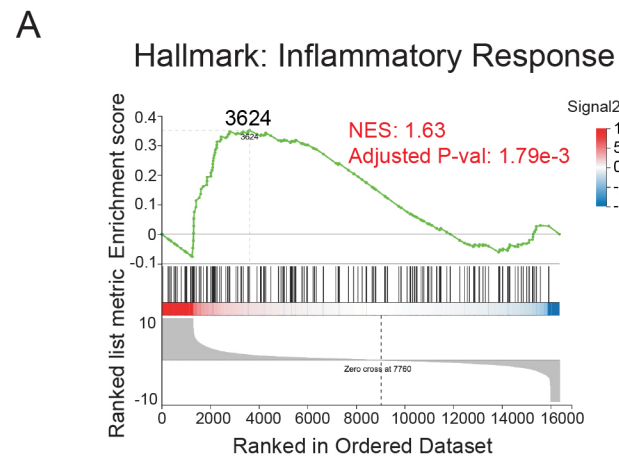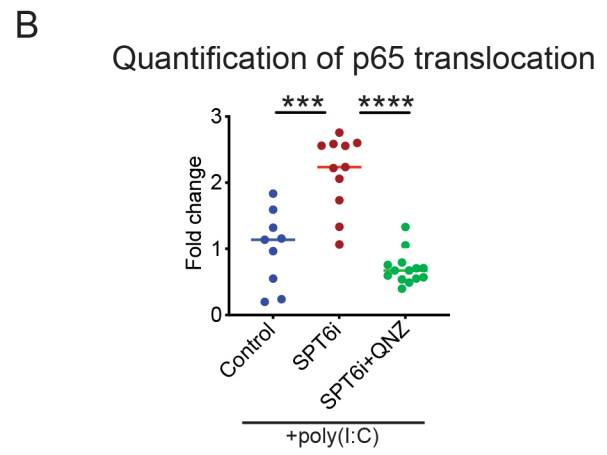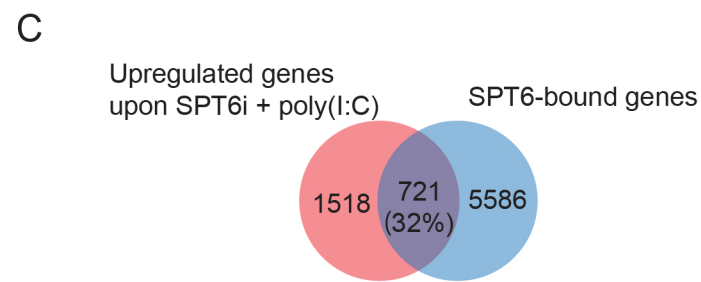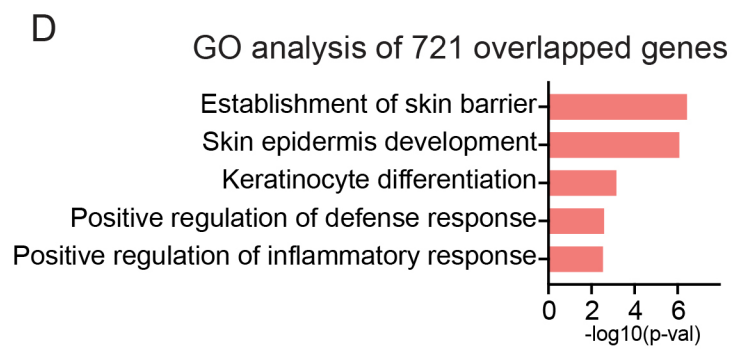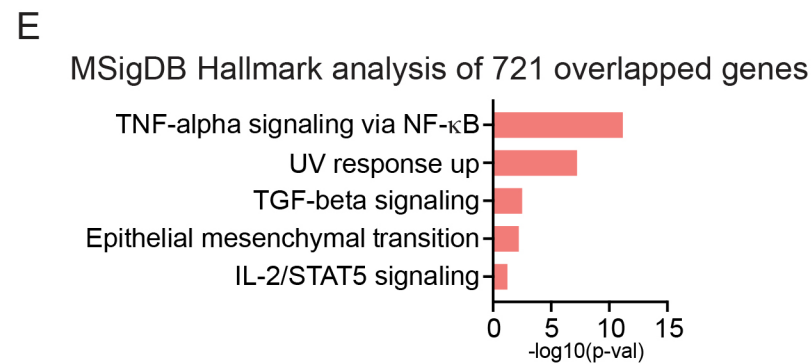

Supplement: Supplementary file 1 — Supplementary Figures 1-8 [file 41423_2026_1410_MOESM1_ESM.pdf]

Fig. 1B

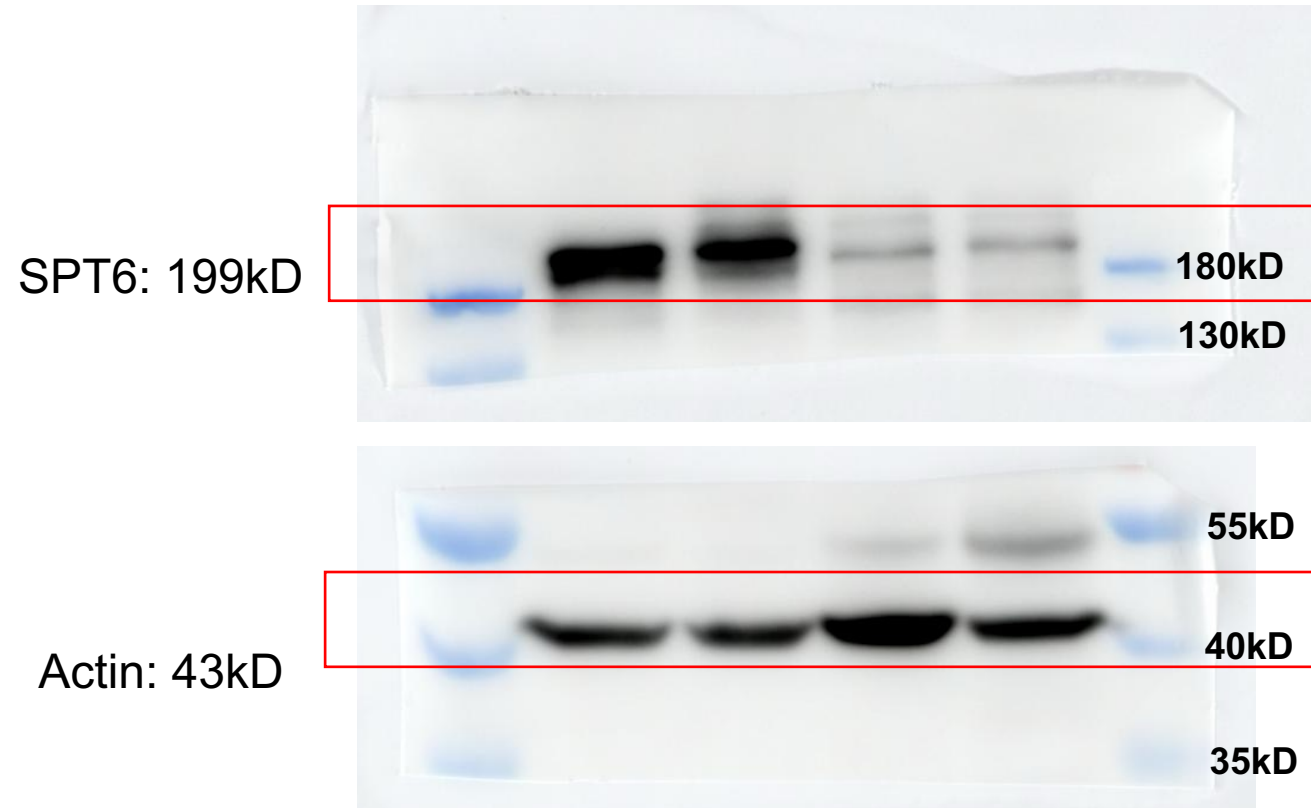

Fig. 7B

SPT6: 199kD

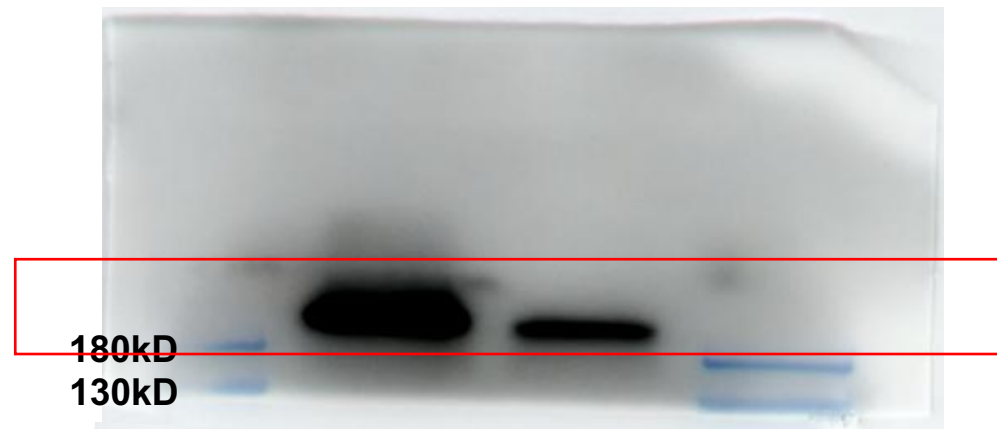

P-p65: 65kD

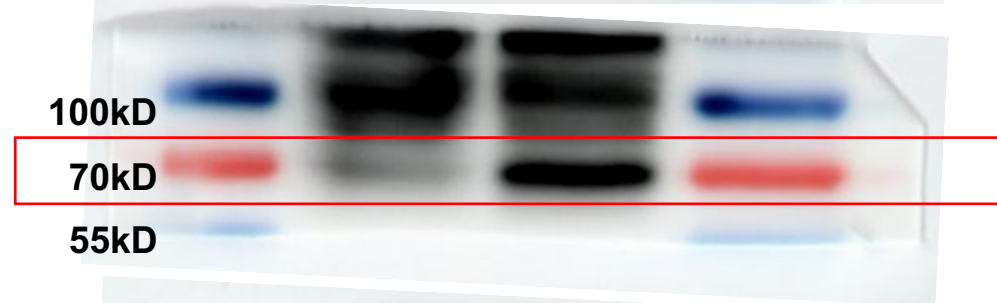

p65: 65kD

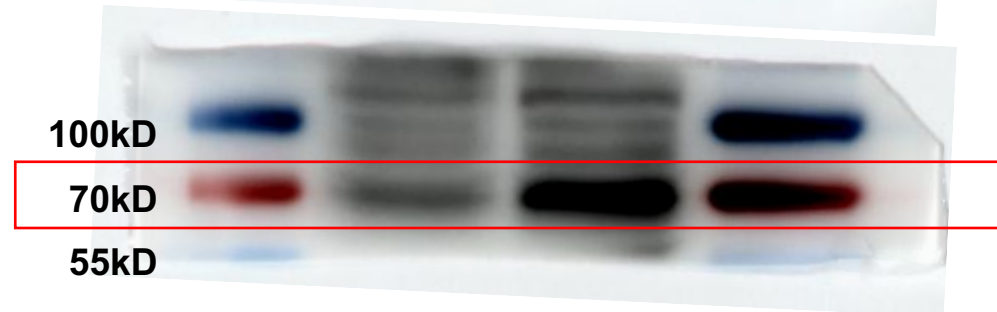

Actin: 43kD

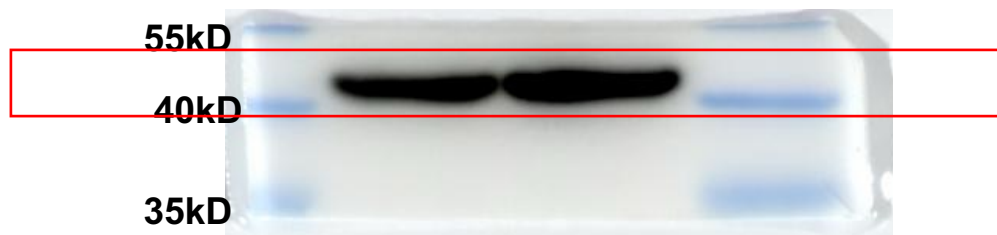

SFig. 1B

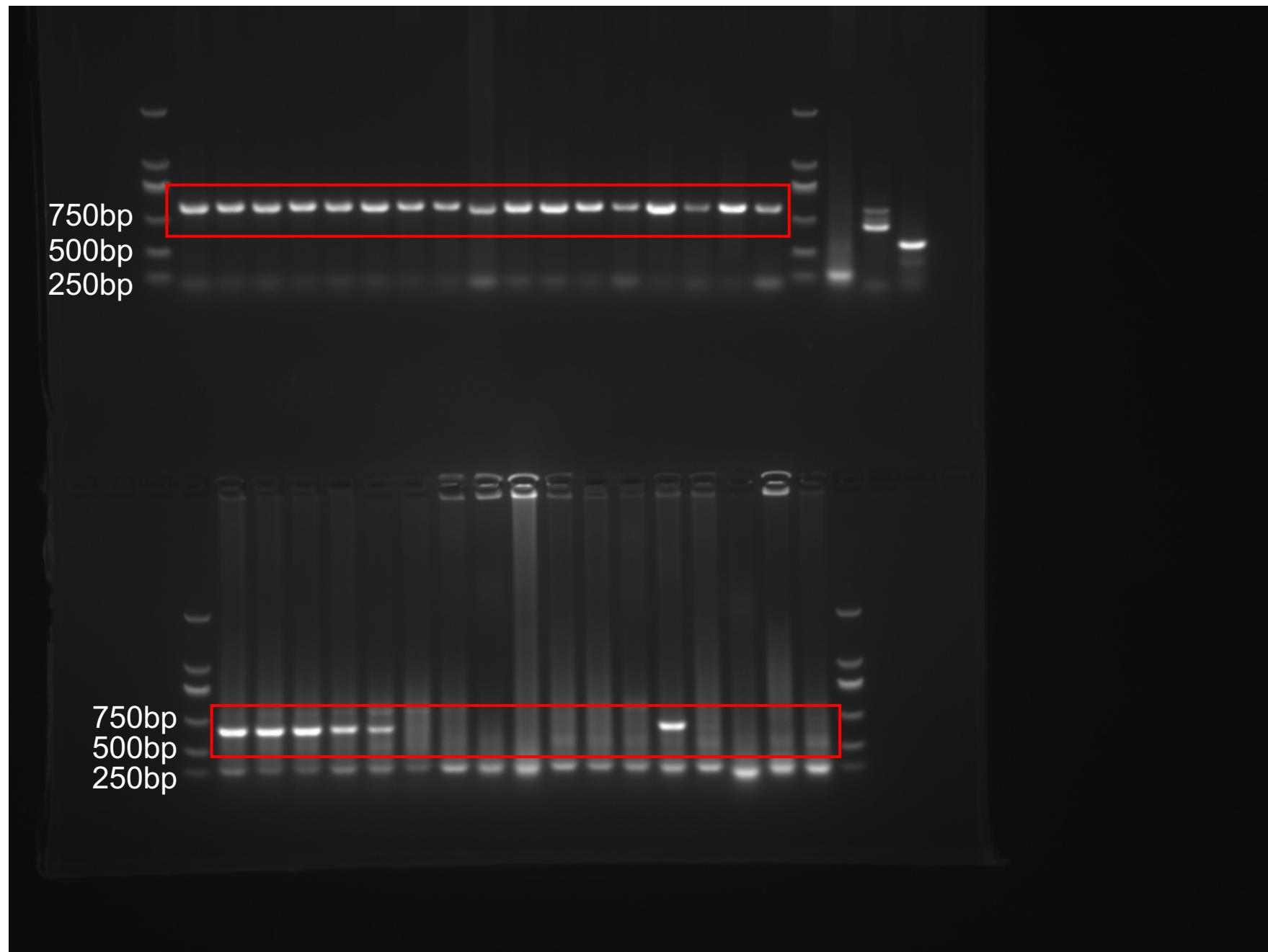

Supplement: Supplementary file 5 — Unprocessed WB images [file 41423_2026_1410_MOESM5_ESM.pdf]
