# Supplementary material for: SPT6 maintains epidermal homeostasis by inhibiting an NF-κB-positive feedback loop to prevent excessive inflammation
Source: Cell Mol Immunol. 2026 Apr 1;23(5):471–90. doi: 10.1038/s41423-026-01410-1 (PMC13129108; doi:10.1038/s41423-026-01410-1)
Supplement: Supplementary file 6 — Supplementary Information [file 41423_2026_1410_MOESM6_ESM.docx]

**Supplementary** **Fig. 1. Construction and validation of epidermal-specific *Supt6* knockout mice.**

(A) PCR analysis of genomic DNA from various tissues confirming tissue-specific SPT6 deletion in floxed and recombinant *Supt6*-KO mice. The upper band (618 bp) corresponds to the floxed *Supt6* allele, whereas the lower band (457 bp) represents the recombined (deleted) allele, indicating successful *Cre*-mediated excision.

(B) H&E-stained cross-sections of tongues (top panel) and esophagi (bottom panel) from control and *Supt6*-KO mice harvested at 10 days after the final tamoxifen injection. The scale bar represents 600 μm (tongue) or 200 μm (esophagus) for H&E staining.

(C) Immunostaining of SOX9, cleaved caspase-3, and Ki67 in the dorsal skin of control and *Supt6*-KO mice harvested on day 0 (telogen) and day 4 (early anagen) after the final tamoxifen injection. The white dashed line denotes hair follicle boundaries.

(D&E) Immunostaining and quantification of Ki67 in the dorsal skin of control and *Supt6*-KO mice harvested on day 7 after the final tamoxifen injection. The white dashed line denotes the epidermis/dermis boundaries.

(F) Representative images showing the appearance of palmar and perianal skin in *Supt6* KO mice at 10 days after the final tamoxifen injection.

(G) H&E staining of ear and digital skin from control and *Supt6*-KO mice harvested at 10 days after the final tamoxifen injection. The scale bar represents 200 μm for H&E staining.

The mean values are shown with error bars representing the SDs. Each dot in the graph represents a single replicate or data from an individual sample. Statistical significance is indicated as follows: ****p <0.0001 (t tests were performed for comparisons between two groups). n ≥ 6 per group

**Supplementary Fig. 2. Characterization of epidermal *Supt6* knockout in adult mice.**

(A) TEM images of dorsal skin from control (left) and *Supt6*-KO (right) mice harvested at 10 days after the final tamoxifen injection. The red asterisk indicates the epidermal side of the skin, and the purple arrowheads indicate the keratohyaline granules.

(B) Quantification of epidermal thickness, relative hemidesmosome length, relative desmosome length, relative keratohyalin granule area, and the nucleolus/nucleus area ratio of the TEM images in (A) and Fig. 2B.

(C) Schematic diagram of tamoxifen-induced SPT6 deletion and time points for skin sample collection in adult mice.

(D) Representative images showing the gross appearance of control and *Supt6* knockout (KO) mice at 10 days after the final tamoxifen injection.

(E) Hematoxylin and eosin (H&E) staining of dorsal and ear skin from control and *Supt6*-KO mice harvested at 10 days after the final tamoxifen injection. The scale bar represents 200 μm for H&E staining.

The mean values are shown with error bars representing the SDs. Each dot in the graph represents a single replicate or data from an individual sample. Statistical significance is indicated as follows: **p <0.01, ***p <0.001, and ****p <0.0001 (one-way ANOVA followed by Tukey’s multiple comparison test for 3 groups and a t test for comparisons between two groups). n ≥ 6 per group.

**Supplementary Fig. 3. Enhanced epidermal differentiation in *Supt6* knockout mouse skin.**

(A&B) Immunostaining and quantification of KRT1 in the dorsal skin of control and *Supt6*-KO mice harvested at 15 and 20 days after the final tamoxifen injection.

(C&D) Immunostaining of KRT1 and quantification of control and *Supt6*-KO ear and tail skin harvested at 10 days after the final tamoxifen injection.

(E&F) Immunostaining of KRT10 and quantification of control and *Supt6*-KO ear and tail skin harvested at 10 days after the final tamoxifen injection.

(G&H) Immunostaining and quantification of LOR in control and *Supt6*-KO epidermis from ear and tail skin harvested at 10 days after the final tamoxifen injection.

(I&J) Immunostaining of IVL and quantification of control and *Supt6*-KO ear and tail skin harvested at 10 days after the final tamoxifen injection.

(K&L) Immunostaining of FLG and quantification of control and *Supt6*-KO ear and tail skin harvested at 10 days after the final tamoxifen injection.

(M&N) Immunostaining and quantification of LOR in control and *Supt6*-KO ventral and dorsal tongue skin harvested at 10 days after the final tamoxifen injection. The white dashed line denotes the epidermis/dermis boundaries. The scale bar represents 50 μm for immunostaining. Statistical significance is indicated as follows: *p <0.05, **p <0.01, ***p <0.001, and ****p <0.0001 (t tests were performed for comparisons between two groups). n ≥ 5 per group.

**Supplementary Fig. 4. Immunostaining of epidermal differentiation markers in adult *Supt6* knockout mouse skin.**

(A&B) Immunostaining and quantification of KRT1 and LOR in adult control and *Supt6*-KO dorsal skin collected at 10 days after the final tamoxifen injection.

(C&D) Immunostaining and quantification of KRT1 and LOR in adult control and *Supt6*-KO ear skin collected at 10 days after the final tamoxifen injection. The white dashed line denotes the epidermis/dermis boundaries. The scale bar represents 50 μm for immunostaining. Statistical significance is indicated as follows: ****p <0.0001 (t tests were performed for comparisons between two groups). n ≥ 5 per group.

**Supplementary Fig. 5. Increased expression of inflammatory genes in *Supt6* knockout mice.**

(A) GSEA indicating the upregulation of immune response pathways in the *Supt6*-KO mice. NES at 1.73, adjusted *p* value at 8.04E-06.

(B) H&E staining of dorsal and ear skin and MPO staining of ear skin from control and *Supt6*-KO mice collected at 10 days after the final tamoxifen injection. The yellow arrowhead indicates epidermal hyperplasia. The green arrowhead indicates Munro’s microabscesses. The black arrowhead indicates hyperkeratosis. The scale bar represents 200 μm for H&E and MPO staining.

(C) Pearson correlation analysis of *Supt6* KO RNA-Seq data with published RNA-Seq profiles (GSE121212) from lesion skin of psoriasis (PSO) patients compared with that of healthy controls. R=0.49, *p* < 0.0001.

(D) Venn diagram showing the overlap between differentially expressed genes (DEGs) from *Supt6*-KO skin (red circle) and day 6 post-IMQ-treated skin RNA-Seq data from GSE92967 (blue circle).

(E) Ridge plot of the results of the KEGG pathway enrichment analysis for the 798 overlapping genes. The X-axis indicates the log2-fold change (FC) in FPKM. Each ridge represents a specific KEGG pathway, with color intensity reflecting the adjusted *p* value, where deeper red indicates stronger statistical significance.

(F) Ridge plot of the five significantly enriched GO terms among the 798 overlapping genes. The X-axis indicates the log2FC of FPKM. Each ridge represents a specific GO term, with color intensity reflecting the adjusted *p* value, where deeper red indicates stronger statistical significance.

(G) Schematic diagram of tamoxifen-induced SPT6 deletion, IMQ treatment timeline, and time points for skin sample collection.

(H) Periodic acid-Schiff (PAS) staining of skin sections from control and *Supt6*-KO mice harvested 10 days after the final tamoxifen injection.

(I) RT‒qPCR analysis of fungal internal transcribed spacer (ITS) sequences in skin scrapings from control and *Supt6*-KO mice.

(J) Fungal colonies were cultured from skin swabs of control and *Supt6*-KO mice on SDA (top left panel) and PDA (bottom left panel) plates, and the quantification of colony numbers is shown in the right panel.

(K) RT‒qPCR analysis of viral DNA in skin samples from control and *Supt6*-KO mice.

(L) H&E staining of dorsal skin harvested from *Supt6*-KO mice with microbiota transfer (from control or *Supt6*-KO mice). The scale bar represents 200 μm for H&E staining.

The mean values are shown with error bars representing the SDs. Each dot in the graph represents a single replicate or data from an individual sample. (A t test was performed for comparisons between two groups). n.s., not significant. n ≥ 4 per group. SDA, Sabouraud dextrose agar; PDA, potato dextrose agar.

**Supplementary Fig. 6. Cell cycle dysregulation in *Supt6* KO epidermis.**

(A) t-Distributed Stochastic Neighbor Embedding (t-SNE) plot of cell types in control and *Supt6*-KO mouse epidermis (n=1 from each group). A total of 7,632 cells from the control epidermis and 10,293 cells from the Supt6-KO epidermis were retained for downstream analyses.

(B) Dot plot showing the expression of representative marker genes associated with the indicated cell types. Dot size indicates the percentage of cells within the subcluster expressing the gene, while color intensity reflects the average expression level, with orange denoting higher expression.

(C) *Supt6* expression across major cell types in control (teal) and *Supt6* KO (red) epidermis.

(D) Bar graph representing the percentage of major cell type populations in control and *Supt6*-KO epidermis.

(E) Cell cycle analysis of control and *Supt6*-KO mouse epidermis. Cells were analyzed for cell cycle distribution and are color-coded by phase: blue for G1, red for G2/M, and green for S phase.

(F) G2/M phase scores in control (teal) and *Supt6*-KO (red) epidermis.

(G) G2/M phase scores in keratinocyte_basal and keratinocyte_suprabasal control (teal) and *Supt6* KO (red) epidermis.

(H) Proportions of cycling cells (G2/M + S phase) in basal and suprabasal keratinocyte populations in control (teal) and *Supt6*-KO (red) epidermis.

(I) *Cdk4* expression in keratinocyte_basal and keratinocyte_suprabasal in control (teal) and *Supt6* KO (red) epidermis.

(J) Dot plot showing the expression of representative genes associated with the indicated subclusters of keratinocytes. Dot size indicates the percentage of cells within the subcluster expressing the gene, while color intensity reflects the average expression level, with orange denoting higher expression.

(K) G2/M phase scores in subclusters 4 (4-Interm-3) and 5 (5-Bas-2) of control (teal) and *Supt6* KO (red) epidermis.

The mean values are shown with error bars representing the SDs. Each dot in the graph represents a single replicate or data from an individual sample. Statistical significance is indicated as follows: *p <0.05, **p <0.01, ***p <0.001, and ****p <0.0001 (one-way ANOVA followed by Tukey’s multiple comparison for 3 groups, and a t test was performed for comparisons between two groups). n ≥ 3 per group.

**Supplementary Fig. 7. Pseudotime trajectory, cell–cell communication, and transcriptional regulatory network analyses revealed altered keratinocyte state transitions upon SPT6 loss.**

(A) Monocle2 pseudotime trajectory analysis of epidermal keratinocyte subclusters. Cells are ordered along pseudotime and colored according to keratinocyte subclusters.

(B) Subset trajectories of subcluster 4 (4-Interm-3) in control and *Supt6* KO epidermis.

(C) Slingshot analysis showing inferred pseudotime density distributions for individual keratinocyte subclusters in control and *Supt6*-KO epidermis.

(D) CytoTRACE analysis showing the predicted differentiation potential of keratinocytes in control and *Supt6*-KO epidermis.

(E) Box plots comparing CytoTRACE scores across keratinocyte subclusters between control (teal) and *Supt6*-KO (red) epidermis.

(F&G) CellPoneDB-based ligand‒receptor interaction analysis of control and *Supt6* KO keratinocyte subclusters. Dot size indicates the -log10(*p* value), while color intensity reflects the average expression level (log2(mean)), with purple indicating higher expression.

(H) Dot plot showing the expression of regulon target genes in associated subclusters. Dot size indicates the percentage of cells within the subcluster expressing the gene, while color intensity reflects the average expression level, with orange denoting higher expression.

Statistical significance is indicated as follows: ****p <0.0001 (a t test was performed for comparisons between two groups).

**Supplementary Fig. 8. SPT6 suppresses inflammation *via* NF-κB signaling in human keratinocytes.**

(A) GSEA indicating the activation of hallmark gene signatures associated with the inflammatory response in SPT6i cells treated with poly(I:C). NES at 1.63, adjusted *p* value at 1.79e-3.

(B) Quantification of nuclear p65 localization in control cells treated with poly(I:C) and in SPT6i cells treated with poly(I:C) alone or in combination with QNZ. The nuclear localization ratio was calculated as the number of p65-positive nuclei divided by the total number of nuclei and is expressed as the fold change over the control.

(C) Venn diagram of the overlap between upregulated genes from SPT6i+Poly(I:C) RNA-Seq (red circle) and SPT6-bound genes (blue circle).

(D) Top enriched GO terms for the 721 overlapping genes according to Enrichr.

(E) Top enriched KEGG pathways for the 721 overlapping genes according to Enrichr
